# Polymerase iota coordinates adaptation to replication stress in BRCA2-deficient mammary epithelial cells

**DOI:** 10.1016/j.isci.2026.117830

**Published:** 2026-10-09

**Authors:** Kavya Vipparthi, Ariana Bellare, Resul Ozbilgic, Peter Bazeley, Nishtha Khanna, Fangfang Wang, Anand Bhushan, Liangqi Xie, Zahraa Al-Hilli, You-Wei Zhang, Mihriban Karaayvaz

**Affiliations:** 1 Department of Cancer Sciences, Cleveland Clinic Research, Cleveland Clinic, Cleveland, OH, USA; 2 Department of Genomic Sciences and Systems Biology, Cleveland Clinic Research, Cleveland Clinic, Cleveland, OH, USA; 3 Department of Biochemistry, School of Medicine, Case Western Reserve University, Cleveland, OH, USA; 4 Department of Biological, Geological, and Environmental Sciences, Cleveland State University, Cleveland, OH, USA; 5 Department of Pharmacology, School of Medicine, Case Western Reserve University, Cleveland, OH, USA; 6 Department of Ophthalmic Research, Cole Eye Institute, Cleveland Clinic Research, Cleveland Clinic, Cleveland, OH, USA; 7 Department of Infection Biology, Cleveland Clinic Research, Cleveland Clinic, Cleveland, OH, USA; 8 Breast Center, Integrated Surgical Institute, Cleveland Clinic, Cleveland, OH, USA; 9 Department of Molecular Medicine, Cleveland Clinic Lerner College of Medicine, Cleveland, OH, USA

**Keywords:** preneoplastic breast biology, BRCA2, polymerase iota, genome instability, epithelial cell state remodeling, breast cancer predispostion

## Abstract

BRCA2 deficiency induces chronic replication stress, yet how human mammary epithelial cells adapt before malignant transformation remains poorly understood. Here, we identify polymerase iota (Pol ι) as a context-dependent regulator of replication stress responses in BRCA2-deficient mammary epithelium. Single-cell transcriptomic analysis of preneoplastic breast epithelium from BRCA2 mutation carriers revealed enrichment of POLI-positive hormone-sensing cells, while BRCA2 depletion increased nuclear Pol ι in mammary epithelial models. Functionally, Pol ι promoted post-S-phase DNA synthesis specifically in BRCA2-deficient cells. Unexpectedly, POLI depletion reduced chromosomal instability and replication stress markers, while enhancing replication fork progression under replication stress. POLI depletion was also associated with expansion of a luminal-like epithelial population, linking altered replication stress processing to epithelial cell state remodeling. These findings identify Pol ι as a regulator of replication stress adaptation and provide a framework for understanding early cellular changes preceding BRCA2-associated breast tumorigenesis.

## Highlights

• BRCA2 carriers show enrichment of Pol ι-positive hormone-sensing luminal epithelial cells
• Pol ι promotes post-S-phase DNA synthesis specifically in BRCA2-deficient cells
• Pol ι loss attenuates replication stress markers in BRCA2-deficient cells
• Pol ι loss is associated with luminal-like epithelial cell state remodeling

## Graphical abstract

**Figure:**
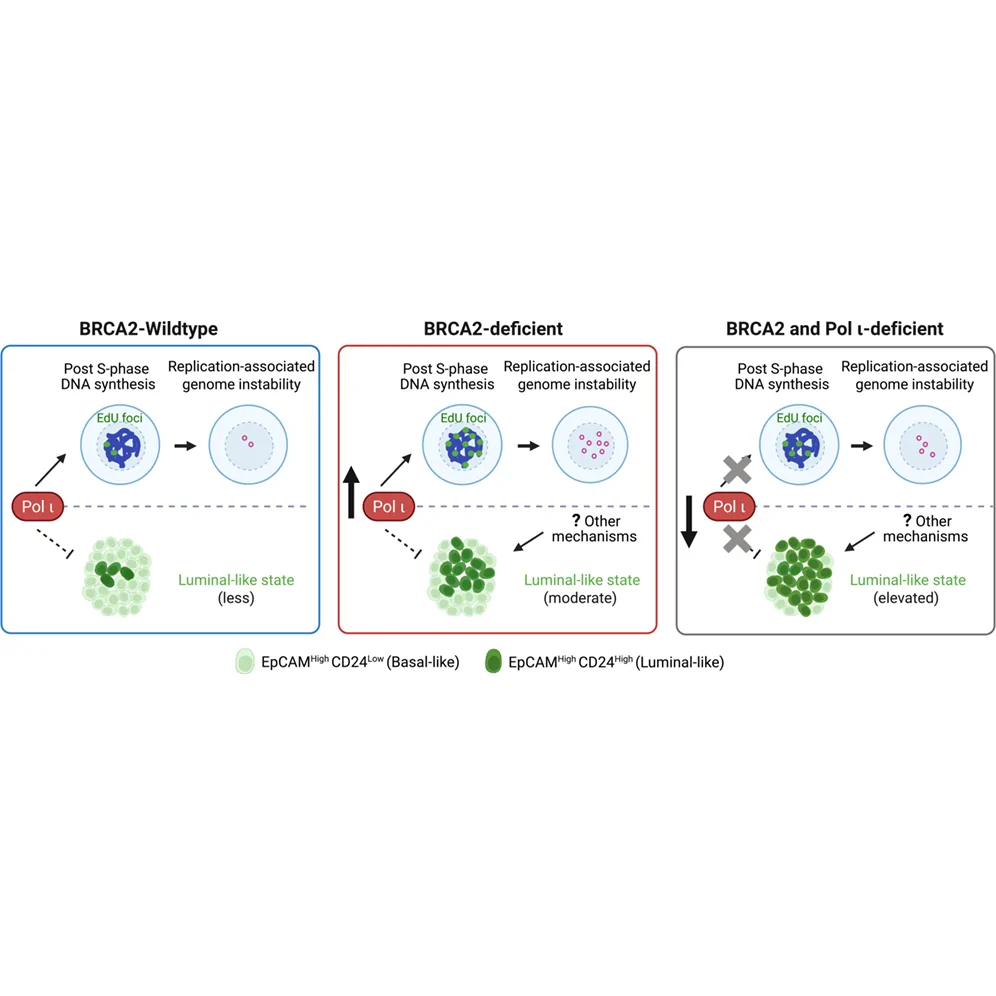

## Teaser

Molecular biology; cell biology; cancer

## Introduction

Germline heterozygous mutations in BRCA2 confer a markedly elevated lifetime risk of breast, ovarian, and prostate cancers.^1^^,^^2^^,^^3^ At the cellular level, BRCA2 plays a central role in preserving genome integrity through multiple mechanisms, including homologous recombination, stabilization of replication forks, and suppression of single-stranded DNA gap accumulation.^4^^,^^5^^,^^6^^,^^7^ Consequently, reduced BRCA2 function results in chronic replication stress, persistence of under-replicated DNA, and progressive genome instability, establishing a permissive state for cancer initiation long before overt malignant transformation.^8^^,^^9^^,^^10^^,^^11^^,^^12^

Importantly, accumulating evidence indicates that breast epithelium from BRCA2 mutation carriers exhibits features consistent with functional haploinsufficiency, such that retention of a single wild-type allele is insufficient to fully maintain genome stability.^13^^,^^14^^,^^15^^,^^16^^,^^17^^,^^18^ In addition to early genomic instability, preneoplastic breast tissues from BRCA mutation carriers exhibit expansion of luminal epithelial populations, indicating that epithelial lineage homeostasis is disrupted during the earliest stages of disease evolution.^19^^,^^20^^,^^21^^,^^22^^,^^23^^,^^24^ Consistent with this notion, we previously demonstrated that non-transformed luminal progenitor cells from BRCA2 mutation carriers harbor sub-chromosomal copy number alterations, indicating that genome instability can arise at very early stages of disease evolution.^25^ Despite these early alterations, the mechanisms by which BRCA2-haploinsufficient epithelial cells adapt to chronic replication stress in primary mammary epithelium remain poorly defined.

One mechanism by which cells cope with unresolved replication stress is the completion of DNA synthesis after S phase. A major form of post-S-phase DNA synthesis is mitotic DNA synthesis (MiDAS), a salvage pathway that completes DNA replication during mitosis.^26^ MiDAS occurs predominantly at common fragile sites, genomic regions that are intrinsically difficult to replicate and highly sensitive to replication stress.^27^^,^^28^ Prior work has identified RAD52 as a core mediator of MiDAS across multiple cellular systems.^29^^,^^30^ Reliance on MiDAS is increased in BRCA2-deficient cells, underscoring its importance as a replication stress-tolerance pathway in this context.^31^^,^^32^^,^^33^ However, the mechanisms that regulate post-S-phase DNA synthesis, including MiDAS, in BRCA2-deficient epithelial cells remain incompletely understood.

Another major pathway by which cells tolerate replication stress is translesion DNA synthesis (TLS), an inherently error-prone DNA damage tolerance pathway mediated primarily by the Y-family polymerases REV1 DNA directed polymerase (REV1), DNA polymerase kappa (Pol κ), DNA polymerase eta (Pol η), and DNA polymerase iota (Pol ι).^34^^,^^35^ These specialized polymerases facilitate DNA synthesis across replication impediments that would otherwise stall replication forks, albeit at the cost of increased mutagenesis.^36^^,^^37^^,^^38^^,^^39^ While TLS pathways have been studied extensively in cancer cells, their contribution to replication stress tolerance in non-transformed, BRCA2-deficient breast epithelium remains largely unexplored.

Recent studies have revealed that specialized DNA polymerases, including REV1 and Pol ζ, contribute to MiDAS,^40^ highlighting a functional intersection between TLS machinery and post-S-phase DNA synthesis. More recently, Pol ι has also been implicated in MiDAS under conditions of replication stress, particularly in transformed cells.^41^ These observations raise the possibility that TLS polymerases may play broader roles in replication stress responses beyond canonical lesion bypass. However, whether Pol ι plays a similar role in the earliest stages of BRCA2-associated breast tumorigenesis, before malignant transformation, remains unknown. Accordingly, the role of Pol ι in coordinating post-S-phase DNA synthesis and replication stress responses in BRCA2-deficient non-transformed mammary epithelial cells has not been investigated.

Because complete BRCA2 loss is incompatible with the viability of non-transformed mammary epithelial cells,^31^ we employed transient BRCA2 silencing to examine how reduced BRCA2 levels remodel replication stress responses in otherwise genomically stable cells. Here, we show that the breast epithelium of BRCA2 mutation carriers exhibits an increased proportion of POLI-positive hormone-sensing (HS) cells, a luminal epithelial population, and that BRCA2 depletion increases Pol ι levels in mammary epithelial cells. Functionally, Pol ι promotes post-S-phase DNA synthesis specifically in BRCA2-deficient mammary epithelial cells. Unexpectedly, despite impairing post-S-phase DNA synthesis, POLI depletion in BRCA2-deficient cells does not exacerbate replication-associated genome instability but instead is associated with increased replication fork progression under replication stress and expansion of a luminal-like EpCAM^high^/CD24^high^ epithelial population. Together, these findings identify Pol ι as a context-dependent regulator of replication stress adaptation in BRCA2-deficient mammary epithelial cells and provide a mechanistic framework linking chronic replication stress to early epithelial alterations that precede BRCA2-associated breast tumorigenesis.

## Results

### BRCA2 deficiency induces POLI in mammary epithelial cells

BRCA2 deficiency generates persistent replication stress that challenges completion of DNA replication.^42^^,^^43^ Because Y-family TLS polymerases facilitate DNA synthesis under conditions of replication stress, we asked whether their expression is altered in mammary epithelial cells from BRCA2 mutation carriers. We analyzed a publicly available single-cell RNA sequencing (scRNA-seq) dataset of primary breast epithelial cells from BRCA2 mutation carriers (BRCA2^mut/+^) and non-carrier controls (BRCA2^+/+^), profiling the expression of the Y-family polymerases REV1, POLK, POLH, and POLI across epithelial cell populations.^44^

Among the four Y-family TLS polymerases examined, POLI showed the most striking genotype-dependent difference. POLI mRNA showed the strongest genotype-dependent increase in HS epithelial cells from BRCA2 mutation carriers, whereas REV1, POLK, and POLH remained unchanged (Figures 1A and 1B). Analysis of the remaining epithelial compartments revealed no genotype-dependent differences in alveolar cells and only a reduction in REV1 expression within basal cells, whereas POLK and POLH remained unchanged (Figures S1A and S1B). Together, these findings identify Pol ι induction as a prominent feature of BRCA2^mut/+^ mammary epithelial cells, rather than reflecting a global upregulation of Y-family TLS genes.

**Figure 1:**
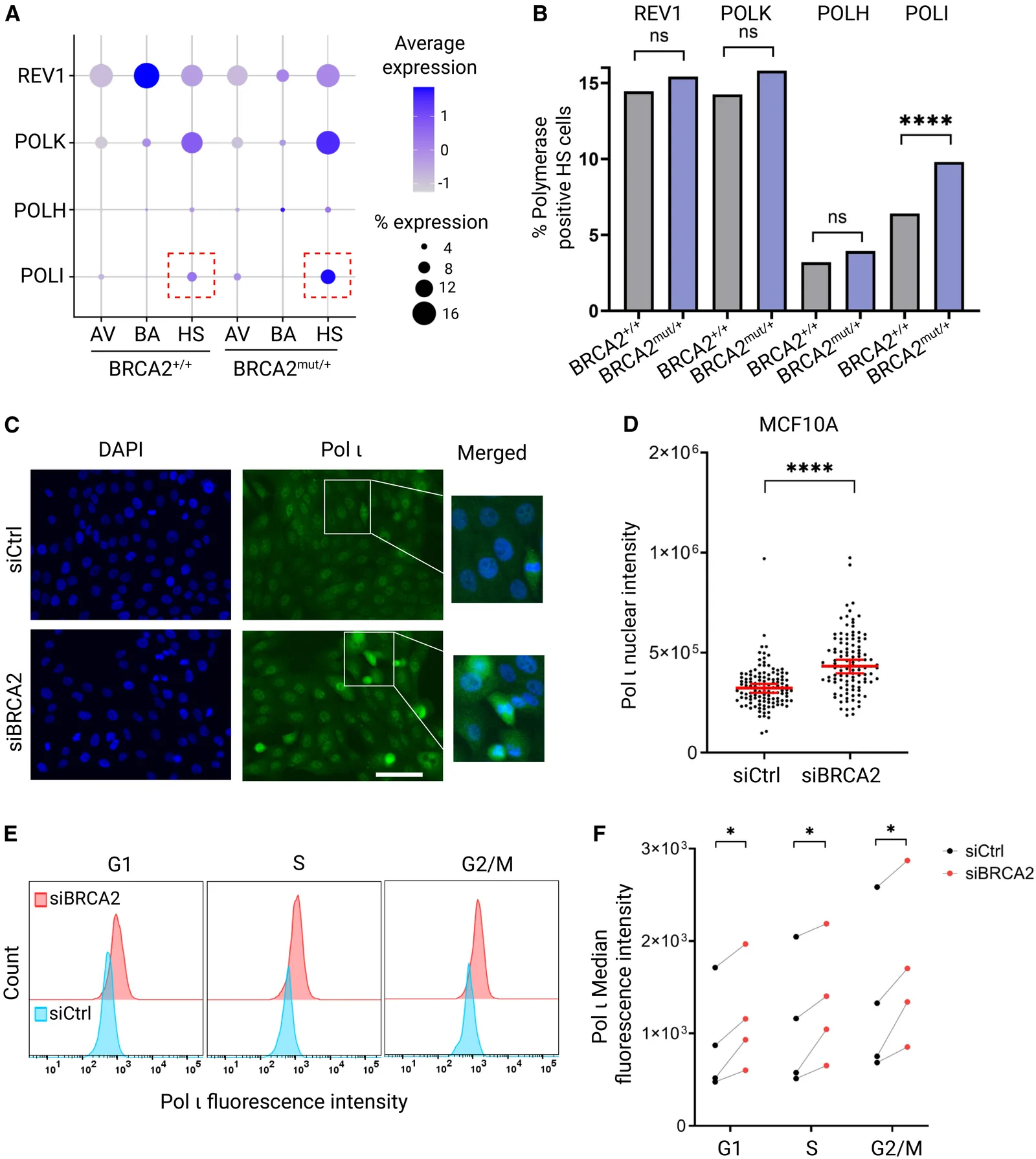
BRCA2 deficiency increases Pol ι expression in mammary epithelial cells (A) Dot plot showing expression of the Y-family polymerases (REV1, POLK, POLH, and POLI) across basal/myoepithelial (BA), alveolar (AV), and hormone-sensing (HS) epithelial populations from a published scRNA-seq dataset of BRCA2^mut/+^ and BRCA2^+/+^ breast epithelium.^44^ Dot size indicates the percentage of cells expressing each polymerase, and color intensity represents relative expression levels (scaled normalized counts). (B) Quantification of the percentage of REV1-, POLK-, POLH-, and POLI-positive HS cells. Pol ι-positive HS cells were selectively enriched among BRCA2^mut/+^ samples. Fisher’s exact test; ∗∗∗∗*p* < 0.0001. (C) Representative immunofluorescence images showing increased nuclear Pol ι (green) following BRCA2 silencing in MCF10A cells. Nuclei were counterstained with DAPI (blue). Scale bars, 75 μm. (D) Quantification of nuclear Pol ι fluorescence intensity in MCF10A cells following BRCA2 silencing. Red lines indicate the median; error bars denote the 95% confidence intervals. Statistical significance was determined using the Mann-Whitney U test; ∗∗∗∗*p* < 0.0001. One representative experiment from three independent biological replicates is shown. Each dot represents a single cell. At least 100 cells per condition were analyzed in each biological replicate. (E) Representative flow cytometry histograms showing increased Pol ι median fluorescence intensity (MFI) in the G1, S, and G2/M phases following BRCA2 silencing in MCF10A cells. (F) Quantification of Pol ι MFI across the G1, S, and G2/M phases from four independent biological experiments. Each dot represents one biological replicate. Statistical significance was determined using a paired two-tailed Student’s *t* test; ∗*p* < 0.05.

To validate the induction of POLI across mammary epithelial cell models, we depleted BRCA2 by small interfering RNA (siRNA) in non-transformed MCF10A cells, primary human mammary epithelial cells (HMECs), and MCF7 breast cancer cells, followed by immunofluorescence analysis of Pol ι protein levels. BRCA2 depletion resulted in a marked increase in the nuclear Pol ι signal in MCF10A cells (Figures 1C and 1D). Similar increases were observed in the HMECs and MCF7 cells (Figures S1C–S1F). Efficient BRCA2 knockdown was confirmed by western blotting in the MCF10A and MCF7 cells (Figures S1G and S1H).

Because BRCA2 deficiency can alter cell-cycle progression, we next asked whether increased Pol ι expression was restricted to specific phases of the cell cycle. Cell cycle analysis revealed only minimal differences between the control and BRCA2-depleted MCF10A cells, with BRCA2 depletion resulting in a slight increase in the proportion of cells in G2/M (Figures S2A and S2B). Combined Pol ι immunostaining with cell cycle analysis demonstrated that BRCA2 depletion increased Pol ι fluorescence intensity across G1, S, and G2/M (Figures 1E, 1F, S2C, and S2D). To determine whether elevated Pol ι expression was preferentially associated with a specific cell cycle phase, we compared the cell-cycle distribution of Pol ι-high and Pol ι-low MCF10A cells. Pol ι-high cells were significantly enriched in G2/M relative to Pol ι-low cells (Figures S2E and S2F).

We next examined whether this enrichment extended to primary human breast epithelial cells by analyzing HS cells from BRCA2^mut/+^ and BRCA2^+/+^ breast tissues. BRCA2^mut/+^ HS epithelial cells exhibited a significantly greater proportion of Pol ι-high cells in S and G2/M than BRCA2^+/+^ HS epithelial cells (Figure S2G). Consistent with these findings, Pol ι-high BRCA2^mut/+^ HS epithelial cells were enriched for mitosis-related transcriptional signatures (Figure S2H), supporting a potential role for Pol ι during late cell-cycle progression. Together, these findings demonstrate that BRCA2 deficiency induces Pol ι in mammary epithelial cells and is associated with the enrichment of POLI-high cells during late cell-cycle progression.

### Pol ι promotes post-S-phase DNA synthesis in BRCA2-deficient cells

BRCA2 deficiency generates regions of under-replicated DNA that are completed through MiDAS.^31^^,^^32^ Because POLI is induced following BRCA2 loss and preferentially accumulates during G2/M, we asked whether Pol ι contributes to post-S-phase DNA synthesis in BRCA2-deficient cells.

To test this, we depleted BRCA2, POLI, or both by siRNA in MCF10A cells, induced mild replication stress with low-dose aphidicolin, and quantified 5-ethynyl-2'-deoxyuridine (EdU) incorporation and FANCD2 foci in phospho-histone H3-positive cells (Figures 2A and 2B), using an established assay.^26^^,^^45^ BRCA2 depletion markedly increased EdU incorporation and FANCD2 foci in phospho-histone H3-positive cells relative to controls, consistent with previous reports.^31^^,^^32^ In contrast, combined depletion of BRCA2 and POLI significantly reduced both readouts toward control levels, whereas POLI depletion alone had little effect (Figures 2C and 2D). These findings were reproduced in MCF7 cells, where BRCA2 depletion increased EdU incorporation and FANCD2 foci in phospho-histone H3-positive cells, whereas combined depletion of BRCA2 and POLI reduced both readouts, and POLI depletion alone had little effect (Figures S3A and S3B).

**Figure 2:**
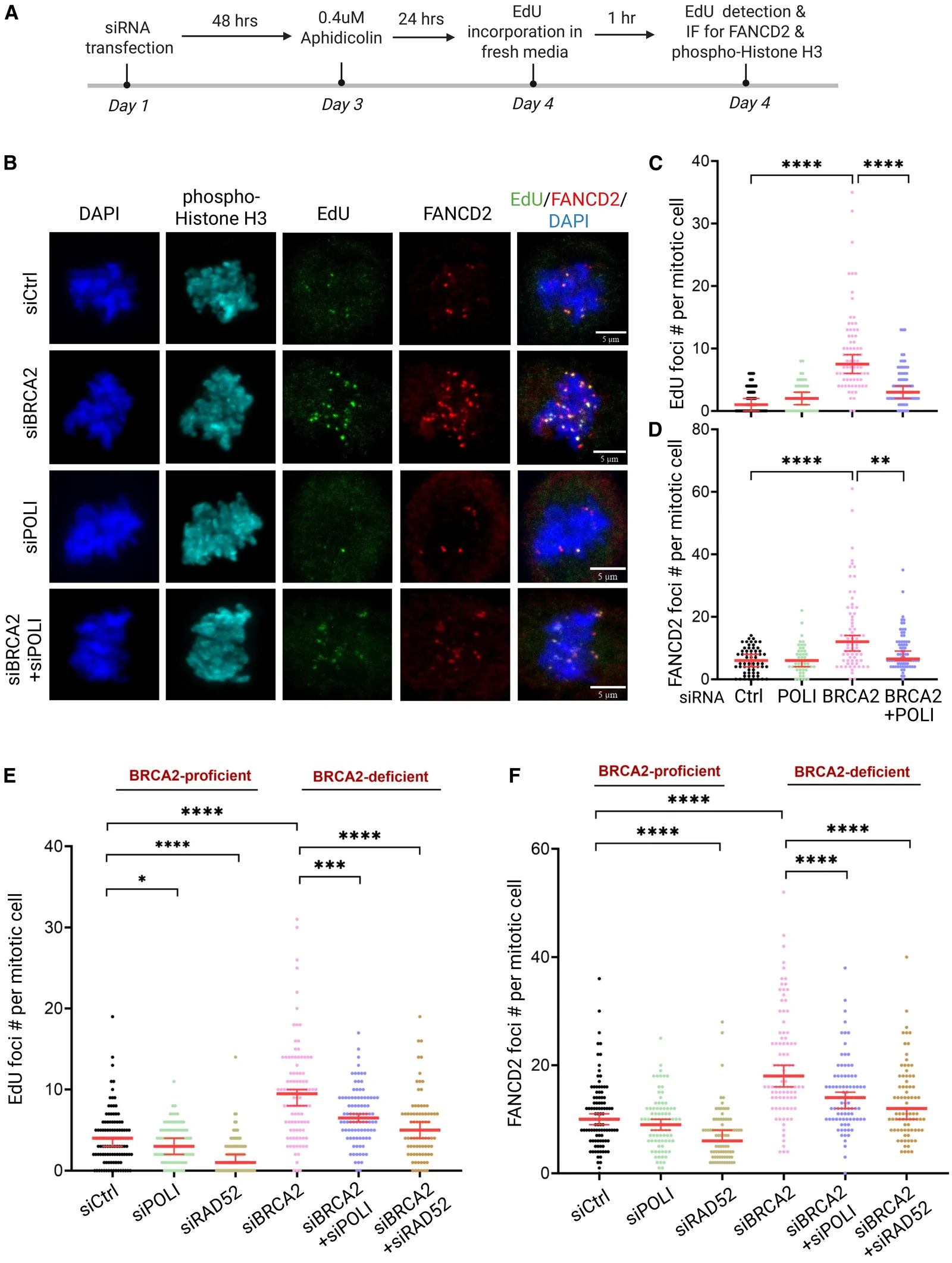
POLI depletion attenuates post-S-phase DNA synthesis in BRCA2-deficient mammary epithelial cells (A) Experimental schematic illustrating the workflow. (B) Representative immunofluorescence images showing EdU incorporation and FANCD2 foci in phospho-histone-H3-positive mitotic MCF10A cells following the indicated siRNA knockdowns. Scale bars, 5 μm. (C–F) Quantification of EdU foci (C and E) and FANCD2 foci (D and F) per mitotic cell for the indicated siRNA knockdowns in MCF10A cells. (C) and (D) compare the control, POLI, BRCA2, and combined BRCA2 + POLI knockdowns, whereas (E) and (F) additionally include RAD52 knockdown conditions. Data represent the pooled values from three (C and D) or two (E and F) independent biological experiments. Approximately 60–80 (C and D) or 90–100 (E and F) mitotic cells were analyzed per condition. Red lines indicate the median; error bars denote the 95% confidence intervals. Statistical significance was determined using the Mann-Whitney U test; ∗*p* < 0.05; ∗∗*p* < 0.01; ∗∗∗*p* < 0.001; ∗∗∗∗*p* < 0.0001.

Given that RAD52 is a canonical mediator of low-dose aphidicolin-induced MiDAS,^29^ we next compared the contribution of Pol ι to that of RAD52. RAD52 depletion alone substantially reduced EdU incorporation, and a similar reduction was observed following co-depletion of BRCA2 and RAD52. In contrast, POLI depletion had little effect on EdU incorporation in BRCA2-proficient cells but markedly reduced EdU incorporation following BRCA2 depletion (Figure 2E). The reduction in EdU incorporation following combined depletion of BRCA2 and POLI was comparable to that observed following combined depletion of BRCA2 and RAD52, although RAD52 produced a stronger overall effect. Consistent with these findings, BRCA2 depletion significantly increased the total number of FANCD2 foci per phospho-histone H3-positive cell, consistent with the accumulation of under-replicated DNA, whereas co-depletion of either POLI or RAD52 significantly reduced FANCD2 foci relative to BRCA2 depletion alone (Figure 2F). Efficient depletion of BRCA2, POLI, and RAD52 was confirmed by western blotting (Figures S2C–S2E). Taken together, these findings identify Pol ι as a previously unrecognized, BRCA2-dependent regulator of post-S-phase DNA synthesis.

### Pol ι loss does not aggravate replication-associated genome instability in BRCA2-deficient cells

BRCA2 deficiency leads to persistent under-replicated DNA that can be detected as 53BP1 nuclear bodies—a widely used marker of replication-associated genome instability—in the subsequent G1 phase.^46^^,^^47^ Because Pol ι promotes post-S-phase DNA synthesis, we next asked whether POLI depletion would increase the persistence of under-replicated DNA and thereby aggravate replication-associated genome instability in BRCA2-deficient cells.

To address this, we quantified 53BP1 nuclear bodies in cyclin A-negative G1 cells following mild replication stress (Figures 3A and 3B). BRCA2 depletion significantly increased 53BP1 nuclear body formation relative to control cells, consistent with increased persistence of under-replicated DNA. Unexpectedly, combined depletion of BRCA2 and POLI did not further increase 53BP1 nuclear body formation and instead showed a trend toward fewer nuclear bodies than BRCA2 depletion alone (Figures 3C and S4A).

**Figure 3:**
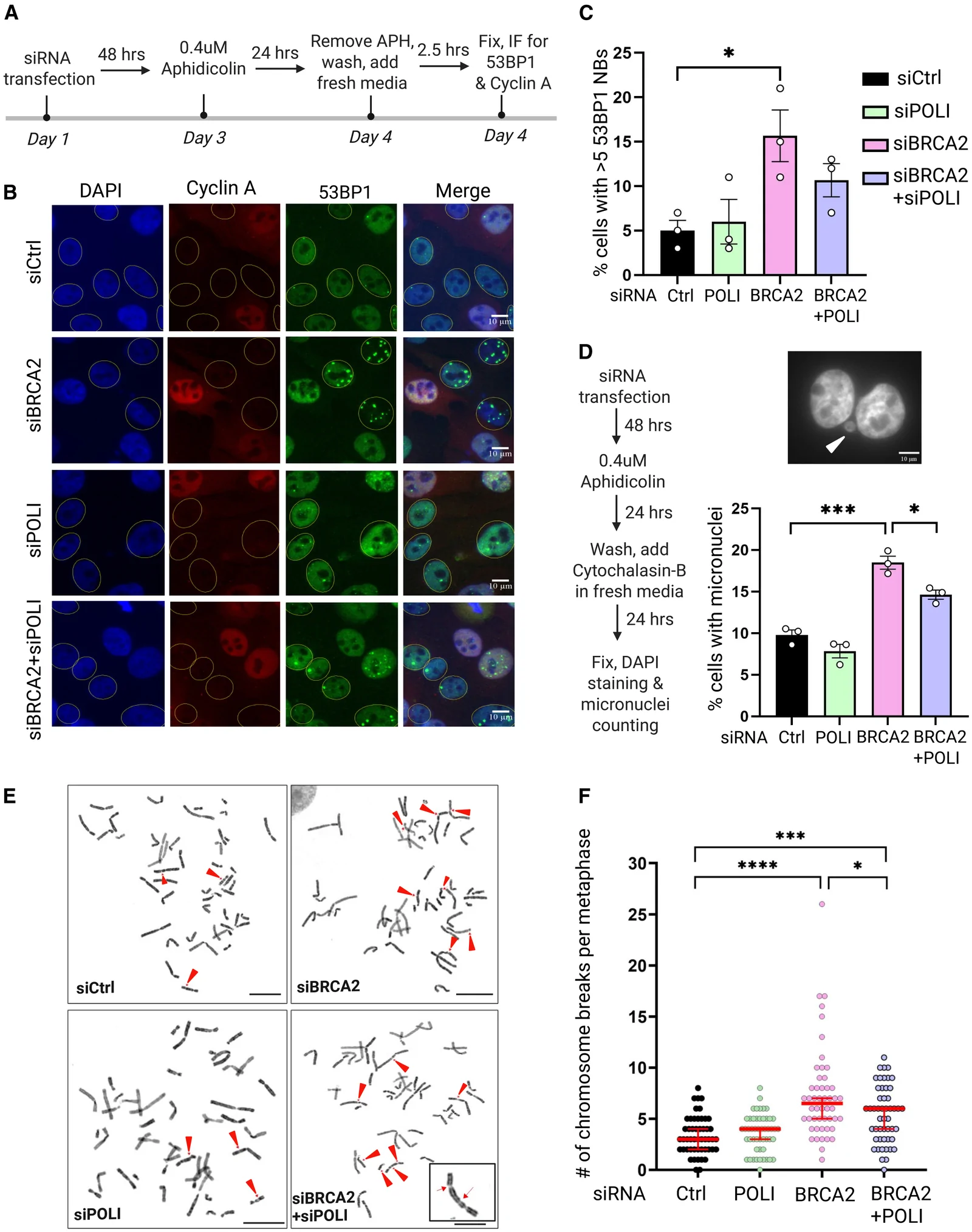
Combined BRCA2 and POLI depletion attenuates replication-associated genome instability (A) Experimental schematic illustrating the workflow for 53BP1 nuclear body (NB) analysis. (B) Representative immunofluorescence images showing 53BP1 NBs in cyclin-A-negative (G1) MCF10A cells following the indicated siRNA knockdowns. Scale bars, 10 μm. (C) Quantification of the percentage of G1 cells containing >5 53BP1 NBs following the indicated siRNA knockdowns. Data represent the mean of three independent biological experiments. Error bars indicate SEM. Statistical significance was determined using a two-tailed unpaired Student’s *t* test; ∗*p* < 0.05. (D) Experimental schematic illustrating the workflow for micronucleus analysis (left). Quantification of the percentage of micronucleus-positive cells following the indicated siRNA knockdowns (right). A representative DAPI-stained nucleus containing a micronucleus is shown. Scale bars, 10 μm. Data represent the mean of three independent biological experiments. Error bars indicate the SEM. Statistical significance was determined using a two-tailed unpaired Student’s *t* test. ∗*p* < 0.05; ∗∗*p* < 0.01; ∗∗∗*p* < 0.001. (E) Representative metaphase spreads following the indicated siRNA knockdowns. Red arrows indicate chromosomal breaks; inset shows a magnified view. Scale bars, 5 μm. (F) Quantification of chromosomal breaks per metaphase following the indicated siRNA knockdowns. Fifty metaphases were analyzed per condition. Red lines indicate the median; error bars denote the 95% confidence intervals. Each dot represents one metaphase. Statistical significance was determined using the Mann-Whitney U test. ∗*p* < 0.05; ∗∗*p* < 0.01; ∗∗∗*p* < 0.001; ∗∗∗∗*p* < 0.0001.

To validate this finding using an independent measure of replication-associated genome instability, we next quantified micronucleus formation. Consistent with prior observations, BRCA2 depletion significantly increased micronucleus formation relative to control cells. Combined depletion of BRCA2 and POLI did not further increase micronucleus formation and instead showed a trend toward fewer micronuclei than BRCA2 depletion alone (Figure 3D). This pattern was reproduced in HeLa cells, where combined depletion of BRCA2 and Pol ι likewise showed a trend toward fewer micronuclei than BRCA2 depletion alone (Figures S4B and S4C).

Because common fragile sites are major sites of MiDAS,^26^^,^^48^ we next analyzed metaphase spreads to determine whether POLI depletion altered chromosome instability at these loci. BRCA2 depletion significantly increased chromosome gaps and breaks relative to control cells. In contrast, combined depletion of BRCA2 and POLI resulted in a modest but statistically significant reduction in chromosome gaps and breaks compared with BRCA2 depletion alone (Figures 3E and 3F).

We next assessed γH2AX as a marker of DNA damage signaling in primary HMECs. Baseline γH2AX levels were higher in BRCA2^mut/+^ cells than in BRCA2^+/+^ controls. Following POLI depletion under mild replication stress, γH2AX levels remained unchanged in BRCA2^+/+^ cells but were significantly reduced in BRCA2^mut/+^ cells (Figure S4D), consistent with reduced replication-associated DNA damage. We also examined RPA32 as a marker of replication stress. RPA32 levels increased following BRCA2 depletion but were significantly reduced following combined depletion of BRCA2 and POLI (Figure S4E). Collectively, these findings demonstrate that POLI depletion does not exacerbate replication-associated genome instability and instead partially attenuates multiple markers of replication stress in BRCA2-deficient cells.

### Pol ι loss enhances replication fork progression under replication stress in BRCA2-deficient cells

Because POLI depletion attenuated multiple markers of replication stress despite reducing post-S-phase DNA synthesis, we next investigated whether replication fork progression was affected in BRCA2-deficient cells. To address this, we performed DNA fiber assays. Cells were sequentially labeled with a short 5-chloro-2′-deoxyuridine (CIdU) pulse (20 min), followed by an extended 5-iodo-2′-deoxyuridine (IdU) pulse (120 min), in the presence of aphidicolin (1.2 μM). Replication fork progression was quantified using the IdU/CIdU tract length ratio. BRCA2 depletion significantly increased the IdU/CIdU ratio, and combined depletion of BRCA2 and POLI resulted in a further significant increase compared with control cells and either single depletion, indicating enhanced replication fork progression under replication stress (Figure 4A). Comparable results were obtained across three independent experiments (Figures S5A and S5B). Parallel untreated controls confirmed that these effects were dependent on replication stress (Figure S5C). Thus, these findings indicate that Pol ι depletion enhances replication fork progression during replication stress in BRCA2-deficient cells, supporting the notion that POLI depletion partially alleviates replication stress despite impairing post-S-phase DNA synthesis.

**Figure 4:**
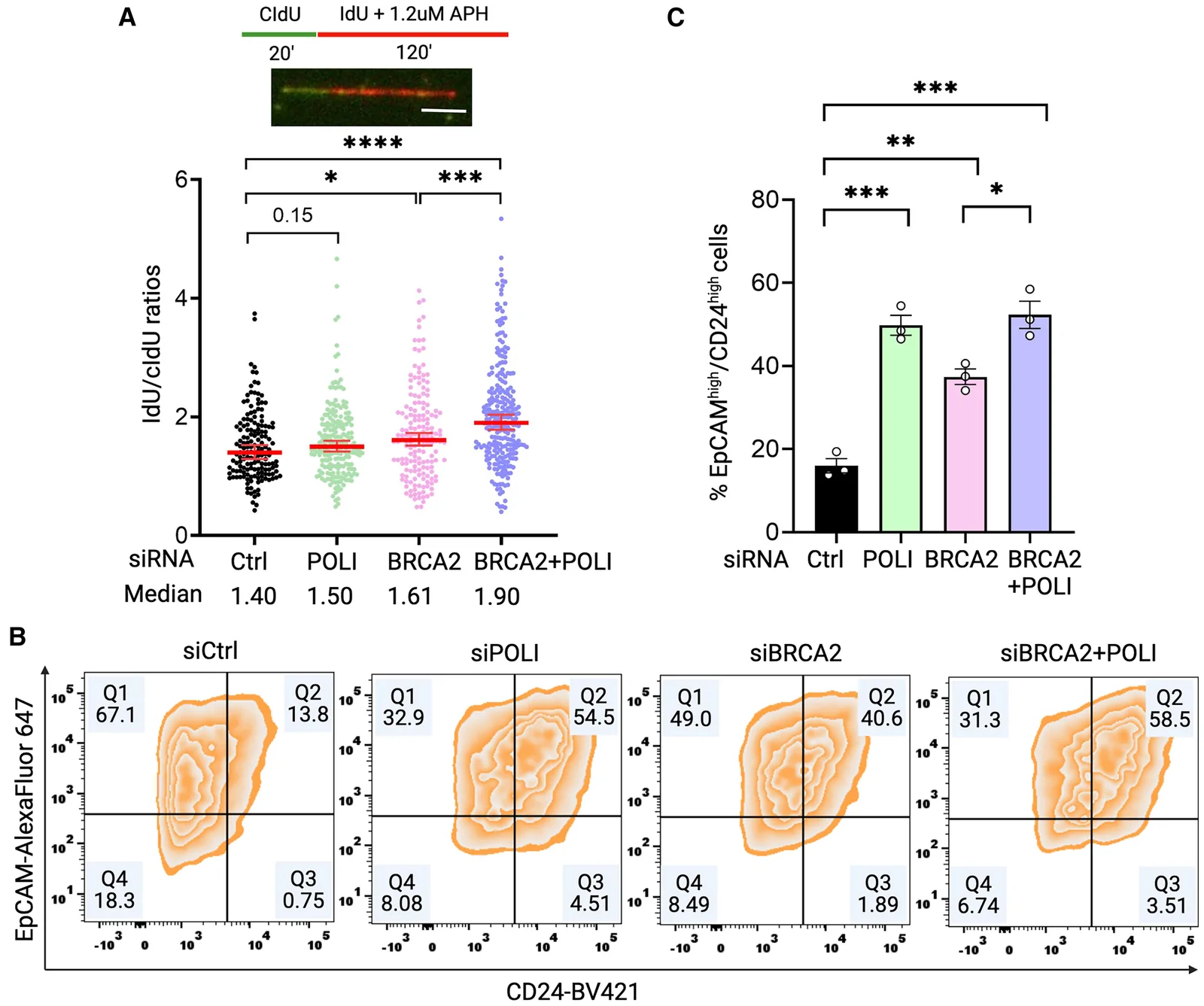
Combined BRCA2 and POLI depletion enhances replication fork progression and is associated with expansion of an EpCAM^high^/CD24^high^ epithelial population (A) Experimental schematic illustrating the DNA fiber assay following aphidicolin treatment (1.2 μM, 2 h) (top). Representative DNA fiber image showing sequential CIdU (green) and IdU (red) labeling. Scale bars, 5 μm. Quantification of IdU/CIdU ratios following the indicated siRNA knockdowns in MCF10A cells. Approximately 150–250 DNA fibers were analyzed per condition. Each dot represents one DNA fiber. Red lines indicate the median; error bars denote the 95% confidence intervals. Median values are shown below each group. Statistical significance was determined using the Mann-Whitney U test. ∗*p* < 0.05; ∗∗*p* < 0.01; ∗∗∗*p* < 0.001; ∗∗∗∗*p* < 0.0001. (B) Representative flow cytometry zebra plots of EpCAM (*y* axis) and CD24 (*x* axis) staining following siRNA knockdowns. (C) Quantification of the percentage of EpCAM^high^/CD24^high^ cells following the indicated siRNA knockdowns. Data represent the mean ± SEM from three independent biological experiments. Statistical significance was determined using a two-tailed unpaired Student’s *t* test. ∗*p* < 0.05; ∗∗*p* < 0.01; ∗∗∗*p* < 0.001; ∗∗∗∗*p* < 0.0001.

### Pol ι loss is associated with acquisition of a stress-adaptive cell state

Our initial analyses identified preferential POLI induction in HS epithelial cells from BRCA2 mutation carriers (Figures 1A and 1B). Because HS cells belong to the luminal epithelial lineage, and expansion of luminal epithelial populations is a well-established feature of preneoplastic breast tissues from BRCA mutation carriers,^19^^,^^20^^,^^21^^,^^22^^,^^23^^,^^24^^,^^25^ we next examined whether BRCA2 or POLI depletion altered the epithelial cell populations in MCF10A cells. We used CD24 as a marker associated with luminal epithelial identity and EpCAM as a pan-epithelial marker.

Flow cytometric analysis demonstrated that both BRCA2 depletion and POLI depletion increased the proportion of EpCAM^high^/CD24^high^ epithelial cells relative to control cells. POLI depletion produced the larger increase, whereas BRCA2 depletion induced a significant but less pronounced expansion of this population. Combined depletion of BRCA2 and POLI did not further increase the proportion of EpCAM^high^/CD24^high^ epithelial cells beyond that observed following POLI depletion alone but remained elevated relative to BRCA2 depletion alone, indicating that the effects of BRCA2 and POLI depletion on this phenotype are not additive (Figures 4B and 4C).

Together, these findings demonstrate that both BRCA2 depletion and POLI depletion promote expansion of the EpCAM^high^/CD24^high^ epithelial population, with POLI depletion producing the stronger effect. Given the established luminal expansion observed in preneoplastic breast tissues from BRCA mutation carriers, these findings are consistent with acquisition of a stress-adaptive luminal epithelial cell state.

## Discussion

Replication stress is a defining feature of BRCA2 deficiency, yet how epithelial cells adapt to persistent replication challenges remains incompletely understood.^49^ In this study, we identify Pol ι as a context-dependent regulator of replication stress responses in BRCA2-deficient HMECs. Our data demonstrated that Pol ι promotes post-S-phase DNA synthesis and, unexpectedly, that POLI depletion attenuated multiple markers of replication-associated genome instability despite impairing post-S-phase DNA synthesis. Together, these findings suggest that POLI depletion remodels how BRCA2-deficient epithelial cells respond to chronic replication stress.

A particularly intriguing finding of this study is the selective increase in the proportion of POLI-positive HS epithelial cells in the breast epithelium of BRCA2 mutation carriers. Because HS cells belong to the luminal epithelial lineage, this observation is particularly noteworthy in light of the well-established expansion of luminal epithelial populations in preneoplastic breast tissues from BRCA mutation carriers. Although the biological significance of this selective enrichment has remained unknown, our functional studies demonstrate that Pol ι promotes post-S-phase DNA synthesis specifically in BRCA2-deficient mammary epithelial cells, suggesting that increased POLI expression within this epithelial population represents a functional adaptation to chronic replication stress in the setting of BRCA2 deficiency.

POLI depletion markedly reduced post-S-phase DNA synthesis in BRCA2-deficient mammary epithelial cells relative to BRCA2 deficiency alone, while having only a minimal effect in BRCA2-proficient cells. These findings indicate that Pol ι is not a universal regulator of post-S-phase DNA synthesis but instead becomes required under conditions of BRCA2-associated replication stress. This context-dependent function suggests that BRCA2-deficient epithelial cells rely on Pol ι to complete DNA synthesis beyond S phase in the presence of replication stress.

Post-S-phase DNA synthesis is generally viewed as a protective mechanism that limits genome instability arising from under-replicated DNA.^26^^,^^50^ Accordingly, loss of Pol ι-mediated post-S-phase DNA synthesis would be expected to exacerbate replication-associated genome instability in BRCA2-deficient cells. Instead, compared with BRCA2 deficiency alone, POLI depletion partially attenuated multiple markers of replication-associated genome instability. Similar attenuation of replication stress-associated damage signaling following POLI loss has been reported previously,^41^ suggesting that this unexpected response to POLI depletion is reproducible across distinct experimental systems.

Analysis of replication fork dynamics provides additional insights into these unexpected findings. DNA fiber assays demonstrated that POLI depletion was associated with increased replication fork progression under replication stress in BRCA2-deficient mammary epithelial cells. Together with the reduction in post-S-phase DNA synthesis, these findings suggest that POLI depletion alters how BRCA2-deficient epithelial cells process replication stress, although the precise mechanisms linking replication fork progression to post-S-phase DNA synthesis remain to be determined. One possible explanation for these observations is that BRCA2-deficient mammary epithelial cells engage compensatory DNA synthesis pathways that sustain replication fork progression despite reduced POLI-dependent post-S-phase DNA synthesis. Defining the molecular basis of this adaptive response requires future investigation.

The observation that POLI depletion altered multiple features of the replication stress response prompted us to investigate whether these adaptive responses were accompanied by changes in the epithelial cell state. Consistent with this possibility, POLI depletion in BRCA2-deficient mammary epithelial cells was associated with expansion of an EpCAM^high^/CD24^high^ epithelial population, suggesting that altered replication stress responses are accompanied by epithelial cell state remodeling. Although BRCA2 deficiency alone increased this population, the larger effect observed following POLI depletion suggests that epithelial cell state remodeling in BRCA2-deficient cells involves both POLI-dependent and POLI-independent adaptive pathways. Previous studies have associated CD24-high epithelial states with adaptive responses to cellular stress in multiple biological contexts.^51^^,^^52^^,^^53^^,^^54^ Together with the well-established expansion of luminal epithelial populations in preneoplastic breast tissues from BRCA mutation carriers, our findings raise the possibility that altered replication stress responses contribute to early epithelial cell state remodeling during BRCA2-associated breast cancer initiation.

Several important questions remain. Although our study establishes a functional role for Pol ι in regulating post-S-phase DNA synthesis and replication fork progression in BRCA2-deficient mammary epithelial cells, the upstream mechanisms responsible for POLI upregulation and the molecular pathways through which Pol ι influences replication fork dynamics remain to be determined. Future studies defining the transcriptional regulation of POLI, its interactions with replication fork remodeling and checkpoint signaling machinery, and the impact of altered replication dynamics on mutational burden will provide further mechanistic insights into these adaptive responses.

Collectively, our findings support a model (graphical abstract) in which BRCA2 deficiency remodels epithelial responses to chronic replication stress through coordinated changes in post-S-phase DNA synthesis, replication fork dynamics, and epithelial cell state. In this model, Pol ι functions as a context-dependent regulator linking replication stress processing with epithelial adaptation in BRCA2-deficient mammary epithelial cells. These findings provide a framework for understanding how early adaptive responses to chronic replication stress shape epithelial evolution during the earliest stages of BRCA2-associated breast tumorigenesis.

### Limitations of the study

Several limitations should be considered when interpreting these findings. First, our functional experiments rely primarily on transient siRNA-mediated silencing, which enables a mechanistic analysis of BRCA2 and POLI functions in non-malignant mammary epithelial cells but captures acute responses. Thus, the consequences of sustained POLI alteration for replication stress adaptation, genome stability, and epithelial cell state remain to be determined. Second, our EdU-based assay detects DNA synthesis occurring across the G2/M boundary but cannot distinguish canonical MiDAS from persistent G2 synthesis or S-to-M runover; accordingly, we refer to these events collectively as post-S-phase DNA synthesis. Finally, although POLI depletion was associated with altered replication stress responses and expansion of a luminal-like epithelial population, our data do not establish a causal relationship between these phenotypes. Determining whether POLI-dependent replication stress processing directly influences epithelial cell state remodeling during BRCA2-associated breast tumorigenesis will require further investigation.

## Resource availability

### Lead contact

Requests for further information and resources should be directed to and will be fulfilled by the lead contact, Mihriban Karaayvaz (karaaym@ccf.org).

### Materials availability

This study did not generate new unique reagents.

### Data and code availability

• All data needed to evaluate the conclusions in the paper are present in the paper or in the supplemental information. The publicly available scRNA-seq dataset reanalyzed in this study is available from the NCBI Gene Expression Omnibus (GEO) under accession GEO: GSE180878.
• This paper does not report original code.
• Any additional information about the data reported in this paper is available from the lead contact upon request.

## Acknowledgments

We sincerely thank Dr. Caroline Astbury and her team -Ms. Rayetta Smith, Mr. Matthew Wolbert, and Ms. Kseniya Budarina- from the Robert J. Tomsich Department of Pathology and Laboratory Medicine Institute, Cleveland Clinic, for assistance with metaphase imaging and analysis. We also thank Dr. Darcie Seachrist and Dr. Ruth Keri for access to resources and Dr. Judith Drazba (Imaging Core, Cleveland Clinic Research) for support with confocal microscopy and imaging consultations. We also thank Flow Cytometry Core, Cleveland Clinic Research, for assistance with flow cytometry experiments. We are grateful to Ms. Tammy Sadler, Mr. Gili Ornan, and Dr. Stephanie Schmit for valuable organizational and resource support. We also thank Mr. Anthony Caputo, formerly a laboratory technician, for technical assistance during the early stages of this work and Dr. Aniket Jana for assistance with microscopy. This work was supported by seed funding from the Cleveland Clinic Research Institute and the American Cancer Society to M.K. and by a Catalyst Grant from Cleveland Clinic to K.V.

## Author contributions

Conceptualization, K.V. and M.K.; methodology, K.V., Y.-W.Z., and M.K.; investigation, K.V., A. Bellare., F.W., A. Bhushan., Y.-W.Z., and M.K.; formal analysis, K.V., A. Bellare., R.O., P.B., N.K., and M.K.; validation, K.V., N.K., and M.K.; data curation, K.V., A. Bellare., and P.B.; writing – original draft, K.V. and M.K.; writing – review & editing, K.V. and M.K.; funding acquisition, K.V. and M.K.; resources, L.X. and M.K.; supervision, M.K.; project administration, M.K.

## Declaration of interests

The authors declare no competing interests.

## Declaration of generative AI and AI-assisted technologies in the manuscript preparation process

During the preparation of this manuscript, the authors used ChatGPT to assist with editing and improve the clarity of the written text. After using this tool, the authors reviewed and edited the content as needed and take full responsibility for the content of the published article.

## STAR★Methods

### Key resources table

**Table:** 

REAGENT or RESOURCE	SOURCE	IDENTIFIER
**Antibodies**		

Rabbit monoclonal anti-FANCD2 (Clone EPR2302)	Abcam	Cat# ab108928, RRID: AB_10862535
Mouse monoclonal anti-phospho-Histone H3 Ser10 (Clone 6G3)	Cell Signaling Technology	Cat# 9706, RRID: AB_331748
Mouse monoclonal anti-Cyclin A2 (Clone 1C6C11)	Proteintech	Cat# 66391-1-Ig, RRID: AB_2881767
Rabbit polyclonal anti-53BP1	Cell Signaling Technology	Cat# 4937, RRID: AB_10694558
Rat monoclonal anti-RPA32/RPA2 (Clone 4E4)	Cell Signaling Technology	Cat# 2208, RRID: AB_2238543
Mouse monoclonal anti-γH2AX Ser139 (Clone JBW301)	Sigma-Aldrich (EMD Millipore)	Cat# 05–636, RRID: AB_309864
Mouse monoclonal anti-DNA Polymerase iota (POLI, Clone OTI1H9)	Abcam	Cat# ab157244
Mouse monoclonal BV421-conjugated anti-CD24 (Clone ML5)	BD Biosciences	Cat# 562789, RRID: AB_2737796
Mouse monoclonal Alexa Fluor 647-conjugated ani-EpCAM (Clone VU1D9)	Cell Signaling Technology	Cat# 5447, RRID: AB_10693458
Rabbit monoclonal anti-BRCA2 (Clone D9S6V)	Cell Signaling Technology	Cat# 10741, RRID: AB_2797730
Rabbit monoclonal anti-RAD52 (Clone EPR3464(2))	Abcam	Cat# ab124971, RRID: AB_10971685
Mouse monoclonal anti-β-Actin (Clone 8H10D10)	Cell Signaling Technology	Cat# 3700, RRID: AB_2242334
Rabbit monoclonal anti-Vinculin (Clone E1E9V)	Cell Signaling Technology	Cat# 13901, RRID: AB_2728768
Mouse monoclonal anti-BrdU (Clone B44)	BD Biosciences	Cat# 347580, RRID: AB_400326
Rat monoclonal anti-BrdU (Clone BU1/75 (ICR1))	Abcam	Cat# ab6326, RRID: AB_305426

**Biological samples**		

Human breast tissues from prophylactic mastectomies of BRCA2 mutation carriers	Tissue Procurement Core, Cleveland Clinic	N/A

**Chemicals, peptides, and recombinant proteins**		

Aphidicolin	Sigma-Aldrich	Cat# 5047440001
Colcemid	Gibco	Cat# 15210
Potassium chloride	Sigma	Cat# P9327
Giemsa	Sigma-Aldrich	Cat# 32884
Cytochalasin-B	Sigma	Cat# C2743
CIdU (5-Chloro-2′-deoxyuridine)	Sigma-Aldrich	Cat# C6891
IdU (5-Iodo-2′-deoxyuridine)	Sigma-Aldrich	Cat# I7125
Lipofectamine RNAiMAX	ThermoFisher Scientific	Cat# 13778075
DAPI	Invitrogen	Cat# P36962
DMEM/F12 media	ThermoFisher Scientific	Cat# 11330057
Horse Serum	ThermoFisher Scientific	Cat# 16050122
Insulin	Sigma	Cat# I-1882
EGF	Peprotech	Cat# AF-100–15
Hydrocortisone	Sigma	Cat# H6909
Cholera toxin	Sigma Aldrich	Cat# C8052
Mammary Epithelial Cell Basal Medium	ATCC	Cat# PCS-600–030,
Mammary Epithelial Cell Growth Kit	ATCC	Cat# PCS-600–040
DMEM/F-12 GlutaMAX	ThermoFisher Scientific	Cat# 10565018
L-glutamine	ThermoFisher Scientific	Cat# 25030081
Gentamycin/amphotericin-B	ThermoFisher Scientific	Cat# R01510

**Critical commercial assays**		

Click-iT EdU Alexa Fluor 488 flow cytometry assay kit	Invitrogen	Cat# C10425
Click-iT EdU Alexa Fluor 488 Imaging kit	Invitrogen	Cat# C10637
FxCycle Violet Ready Flow Reagent	Invitrogen	Cat# R37166
Live-or-Dye 510/551	Biotium	Cat # 32012A
MycoAlert Mycoplasma Detection Kit	Lonza	Cat# LT07–218
Pierce™ Rapid Gold BCA Protein Assay Kit	Thermo Scientific,	Cat# A53225
RIPA buffer	Cell Signaling Technology	Cat# 9803/9806
Protease and phosphatase inhibitors	Cell Signaling Technology	Cat# 5872
PMSF	Cell Signaling Technology	Cat# 8553
SDS-PAGE Precast Protein Gels	Bio-Rad	Cat# 4561084
Clarity Max Western ECL Substrate	Bio-Rad	Cat# 1705062

**Experimental models: Cell lines**		

Human mammary epithelial cell line: MCF10A	Cleveland Clinic Research, cell and media production core facility	Cat# ATCC, CRL-10317
Primary Human Mammary Epithelial Cells: HMECs	American Type Culture Collection (ATCC)	Cat# ATCC, PCS-600–010
Primary BRCA2-mutant Human Mammary Epithelial Cells: BRCA2^mut/-^ HMECs	Tissue Procurement Core, Cleveland Clinic	Caputo et al.^55^
Human mammary cancer cell line: MCF7	Cleveland Clinic Research, cell and media production core facility	Cat# ATCC, HTB-22
Human cervical cancer cell line: HeLa	Cleveland Clinic Research, cell and media production core facility	Cat# ATCC, CRM-CCL-2

**Oligonucleotides**		

siRNA sequences	Dharmacon Horizon Discovery	Supplementary file, this paper

**Software and algorithms**		

R (version 4.5.3)	R Software	https://www.r-project.org/
Seurat (version 5.4.0)	Hao, Y. et al., *Dictionary learning for integrative, multimodal and scalable single-cell analysis.* Nature biotechnology, 2024. 42(2): p. 293–304	https://doi.org/10.1038/s41587-023-01767-y
Scran (version 1.38.0)	Lun, A.T., D.J. McCarthy, and J.C. Marioni, *A step-by-step workflow for low-level analysis of single-cell RNA-seq data with Bioconductor.* F1000Research, 2016. 5: p. 2122	https://doi.org/10.12688/f1000research.9501.2
Scuttle (version 1.20.0)	McCarthy, D.J. et al., *Scater: pre-processing, quality control, normalization and visualization of single-cell RNA-seq data in R.* Bioinformatics, 2017. 33(8): p. 1179–1186	https://doi.org/10.1093/bioinformatics/btw777
Dreamlet (version 1.8.0)	Hoffman, G.E. et al., *Efficient differential expression analysis of large-scale single cell transcriptomics data using dreamlet.* BioRxiv, 2024: p. 2023.03. 17.533005.	https://doi.org/10.1101/2023.03.17.533005
ANUBIX (version 2.0.0)	Castresana-Aguirre, M. and E.L. Sonnhammer, *Pathway-specific model estimation for improved pathway annotation by network crosstalk.* Scientific Reports, 2020. 10(1): p. 13585	https://doi.org/10.1038/s41598-020-70239-z
msigdbr (version 26.1.0)	Dolgalev, I., *msigdbr: MSigDB Gene sets for multiple organisms in a tidy data format. R package version 7.5. 1*. 2022	https://cran.r-project.org/package=msigdbr
ggplot2 (version 4.0.2)	Wickham, H., *Elegant graphics for data analysis*. 2016, Springer. Springer-Verlag New York. ISBN 978-3-319–24277–4.	https://ggplot2.tidyverse.org
sccomp (version 2.1.27)	Mangiola, S. et al., *sccomp: Robust differential composition and variability analysis for single-cell data.* Proceedings of the National Academy of Sciences, 2023. 120(33): p. e2203828120	https://doi.org/10.1073/pnas.2203828120
FunCoup version 6 (https://funcoup.org/download/network&FC6.0_H.sapiens_full.gz)	Buzzao, D. et al., *FunCoup 6: advancing functional association networks across species with directed links and improved user experience.* Nucleic Acids Research, 2025. 53(D1): p. D658–D671	https://doi.org/10.1093/nar/gkae1021
ImageJ	National Institue of Health, USA	ImageJ, https://hpc.nih.gov/apps/Fiji.html
GraphPad Prism Version 11.0.2.(92)	GraphPad	https://www.graphpad.com/

### Experimental model and study participant details

#### Cell lines

MCF10A (ATCC, CRL-10317), MCF7 (ATCC, HTB-22) and HeLa (ATCC, CRM-CCL-2) human cell lines were obtained from CCF Cell and Media Production Core. Cell line identities were authenticated by short tandem repeat (STR) profiling. MCF10A cells were maintained in DMEM/F12 medium (ThermoFisher Scientific, 11330057) supplemented with 5% horse serum (ThermoFisher Scientific, 16050122), 10 μg/mL insulin (Sigma, I-1882), 20 ng/mL human recombinant epidermal growth factor (EGF; Peprotech, AF-100–15), 500 ng/mL hydrocortisone (Sigma, H6909), 100 ng/mL cholera toxin (Sigma Aldrich, C8052), and 50 units/mL penicillin-streptomycin. MCF7 and HeLa were maintained in DMEM medium (ThermoFisher Scientific,11965092) supplemented with 10% fetal bovine serum (ThermoFisher Scientific, A5256701) and penicillin-streptomycin.

#### ATCC HMECs

Commercially available primary HMECs were cultured in Mammary Epithelial Cell Basal Medium supplemented with the Mammary Epithelial Cell Growth Kit according to the manufacturer’s instructions (ATCC, PCS-600–010, PCS-600–030, PCS-600–040).

#### Primary human breast tissues and derived HMECs

Fresh human breast tissues were obtained from two female donors at Cleveland Clinic, including one BRCA2 germline mutation carrier (age 28) and one BRCA2 wild-type control (age 35). BRCA2 germline mutation status was documented in the clinical history. Tissues were obtained under a secondary-use protocol approved by the Cleveland Clinic Institutional Review Board (23–923). Informed consent for tissue collection had been obtained from both participants. Primary HMECs were derived from these tissues as previously described,^55^ and cultured in DMEM/F-12 GlutaMAX (ThermoFisher Scientific, 10565018) supplemented with fetal bovine serum, insulin, EGF, hydrocortisone, L-glutamine (ThermoFisher Scientific, 25030081), penicillin-streptomycin and gentamycin/amphotericin-B (ThermoFisher Scientific, R01510).

All cell cultures were maintained at 37 °C in a humidified 5% CO_2_ incubator and routinely tested for mycoplasma contamination using the MycoAlert Mycoplasma Detection Kit (Lonza, LT07–218).

### Method details

#### siRNA transfections

On-Target plus siRNA SMARTpools were obtained from Dharmacon Horizon Discovery and used for transient gene silencing. Reverse transfections were performed using Lipofectamine RNAiMAX (ThermoFisher Scientific, 13778075) according to the manufacturer’s instructions. A final siRNA concentration of 10 nM was used for all experiments to minimize off-target effects.

#### Immunofluorescence

Cells were seeded on poly-D-lysine -coated chamber slides, reverse transfected as indicated, and processed for immunofluorescence using standard fixation, permeabilization, and blocking conditions. Primary antibodies were incubated overnight at 4 °C, followed by Alexa Fluor-conjugated secondary antibodies. Slides were mounted with antifade reagent containing DAPI (Invitrogen, P36962).

Primary antibodies included: α-POLI (1:250, Abcam Cat# ab157244), α-FANCD2 (1:400, Abcam Cat# ab108928, RRID: AB_10862535), α-phospho-Histone-H3 (1:400, CST Cat# 9706, RRID: AB_331748), α-Cyclin A2 (1:150, Proteintech Cat# 66391-1-Ig, RRID: AB_2881767), α-53BP1 (1:150, CST Cat# 4937, RRID: AB_10694558), α-RPA32 (1:200, CST Cat# 2208, RRID: AB_2238543), α-γH2AX Ser139 (1:200, Sigma-Aldrich Cat# 05–636, RRID: AB_309864). Images were acquired using an EVOS M5000 microscope (ThermoFisher Scientific, A40486) at 40× magnification with identical acquisition settings across conditions.

#### Flow cytometry for cell cycle and Pol ι analysis

Cells were transfected with siRNAs for 72 h, and cell-cycle analysis was performed using the Click-iT EdU Alexa Fluor 488 flow cytometry assay kit (Cat# C10425, Invitrogen) according to the manufacturer’s instructions. Briefly, cells were pulse-labeled with 10 μM EdU for 1 h, harvested, and processed using the Click-iT reaction to fluorescently label incorporated EdU. For Pol ι staining, cells were incubated with α-POLI primary antibody (1:50–1:100, Abcam Cat# ab157244) for 45 min at RT, washed and incubated with Alexa Fluor 647 conjugated secondary antibody (1:2000, Cat # A21235, Invitrogen) for 30 min at RT. Samples were washed twice, and DNA was counterstained with the FxCycle violet dye (Cat# R37166, Invitrogen). Samples were acquired on a BD FACSymphony Symphony A5 flow cytometer. Cell cycle distribution (G1, S, and G2/M phases) was determined based on DNA content and EdU incorporation using FlowJo software (BD Biosciences).

#### Flow cytometry for cell surface markers

Cells were transfected with siRNAs for 72 h, and single cell suspensions were prepared using Accutase cell dissociation reagent (Cat. # A1110501; Thermo) and washed twice with PBS. For live/dead cell discrimination, cells were incubated with Live-or-Dye 510/551 (1:1000, Biotium Cat # 32012A) for 30 min on ice. Cells were then washed with FACS staining buffer (PBS with 0.5% BSA) and incubated with BV421-conjugated α-CD24 antibody (1:50, BD Biosciences Cat# 562789, RRID: AB_2737796) and Alexa Fluor 647-conjugated α-EpCAM antibody (1:50, Cell Signaling Technology Cat# 5447, RRID: AB_10693458) in FACS staining buffer for 30 min on ice. After two washes, cells were fixed with 4% Paraformaldehyde for 15 min at RT, washed twice with PBS, acquired on BD FACSymphony A5 flow cytometer, and analyzed using FlowJo software (BD Biosciences).

#### Western blotting

The siRNA-mediated knockdown of BRCA2, POLI and RAD52 was validated by Western blotting. Cells were lysed in RIPA buffer (CST, 9803/9806) supplemented with protease and phosphatase inhibitors (CST, 5872) and PMSF (CST, 8553), and protein concentrations were determined by BCA assay (Thermo Scientific, A53225). Equal amounts of total protein were resolved by SDS-PAGE (Bio-Rad, 4561084), transferred to PVDF membranes (Bio-Rad, 1620177), and probed with the following antibodies: α-BRCA2 (1:1000, CST Cat# 10741, RRID: AB_2797730), α-POLI (1:2000, Abcam Cat# ab157244, ABclonal Cat# A1942, RRID: AB_2763968), α-RAD52 (1:1000, Abcam Cat# ab124971, RRID: AB_10971685), α-β-Actin (1:2000, CST Cat# 3700, RRID: AB_2242334) and α-Vinculin (1:2000, CST Cat# 13901, RRID: AB_2728768). Proteins were detected by chemiluminescence (Bio-Rad, 1705062) and imaged using an Amersham Imager 600 system (GE Healthcare).

#### Edu incorporation and imaging

Cells were transfected with siRNAs for 48 h and treated with aphidicolin (0.4 μM) for 24 h prior to EdU labeling (10 μM, 1h). EdU incorporation was detected using the Click-iT EdU Imaging Kit (Invitrogen, C10637) according to the manufacturer’s instructions. Following EdU detection, cells were immunostained for phospho-Histone H3 and FANCD2 as described above. Images were acquired using EVOS M5000, Leica SP8 confocal and Zeiss AxioObserver microscopes (lattice SIM) at 40× and 63× magnification.

#### DNA fiber assay

DNA fiber assays were performed using sequential CIdU and IdU labeling to assess replication fork dynamics, as outlined in the experimental schematic. Cells were pulse-labeled with CIdU (100 μM, 20 min) followed by ldU (250 μM, 120 min) in the presence of aphidicolin (0.4 μM). Parallel untreated control samples underwent the same labeling protocol in the absence of aphidicolin.

Following labeling, cells were harvested and DNA fibers were spread onto glass slides under alkaline lysis conditions, For immunofluorescence-based detection of incorporated nucleoside analogs, DNA fibers were denatured with acid and incubated with BrdU-specific primary antibodies to detect CIdU (mouse α-BrdU, BD Biosciences Cat# 347580, RRID: AB_400326) and IdU (rat α-BrdU, Abcam Cat# ab6326, RRID: AB_305426), followed by Alexa Fluor-conjugated secondary antibodies. Slides were mounted using antifade mounting medium containing DAPI.

DNA fibers were visualized using either an EVOS M5000 widefield fluorescence microscope or a Leica Stellaris 5 confocal microscope at 40× and 63× magnification, respectively. Fiber track lengths were quantified using ImageJ software (v1.52q) following calibration with reference scale bars acquired under identical imaging conditions. CIdU and IdU tract lengths were measured independently for each fiber.

### Quantification and statistical analysis

Statistical analyses were performed using GraphPad Prism software. For pairwise comparisons, two-tailed Student’s *t*-tests were used for normally distributed data, whereas the Mann–Whitney *U* tests were used for non-normally distributed data. The statistical tests used, the number of independent biological replicates, and sample sizes are indicated in the corresponding figure legends. Statistical significance was defined as *p* < 0.05 and is indicated as follows: ∗*p* < 0.05; ∗∗*p* < 0.01; ∗∗∗*p* < 0.001; ∗∗∗∗*p* < 0.0001.

## Supplemental information

Document S1. Figures S1–S5 and supplemental methods and references
