# Supplementary material for: Polymerase iota coordinates adaptation to replication stress in BRCA2-deficient mammary epithelial cells
Source: iScience. 2026 Oct 9;29(11):117830. doi: 10.1016/j.isci.2026.117830 (PMC13677199; doi:10.1016/j.isci.2026.117830)
Supplement: Supplementary file 1 [file mmc1.pdf]

iScience, Volume *29*

Supplemental information

Polymerase iota coordinates adaptation to replication stress in BRCA2-deficient mammary epithelial cells

Kavya Vipparthi, Ariana Bellare, Resul Ozbilgic, Peter Bazeley, Nishtha Khanna, Fangfang Wang, Anand Bhushan, Liangqi Xie, Zahraa Al-Hilli, You-Wei Zhang, and Mihriban Karaayvaz

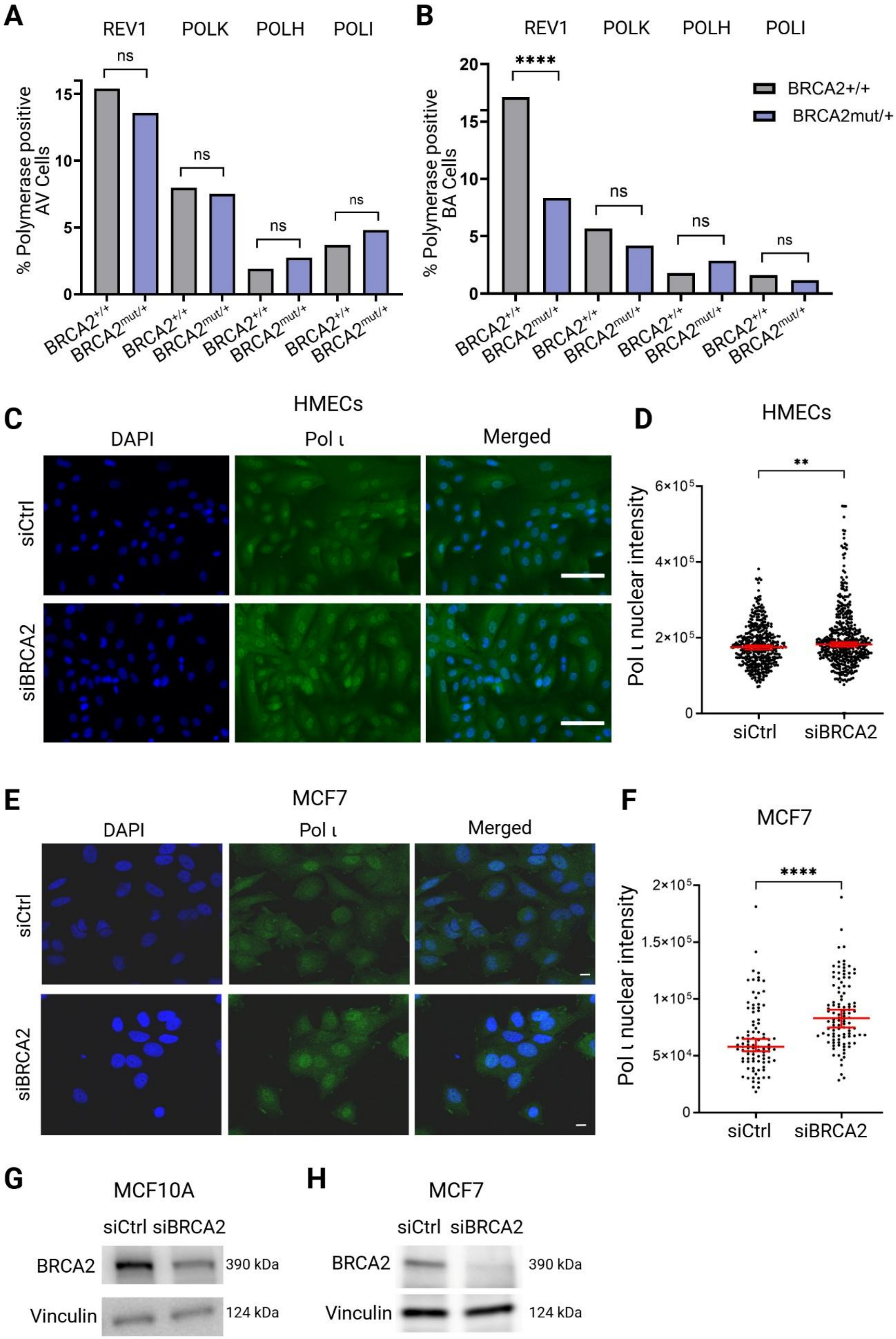
A
REV1
POLK
POLH
POLI
% Polymerase positive AV Cells
ns
BRCA2+/+
BRCA2mut/+
B
% Polymerase positive BA Cells
****
BRCA2+/+
BRCA2mut/+
C
HMECs
DAPI
Pol ι
Merged
siCtrl
siBRCA2
D
HMECs
Pol ι nuclear intensity
**
siCtrl
siBRCA2
E
MCF7
DAPI
Pol ι
Merged
siCtrl
siBRCA2
F
MCF7
Pol ι nuclear intensity
****
siCtrl
siBRCA2
G
MCF10A
siCtrl siBRCA2
BRCA2
390 kDa
Vinculin
124 kDa
H
MCF7
siCtrl siBRCA2
BRCA2
390 kDa
Vinculin
124 kDa

Supplementary Figure S1. POLI is not increased in alveolar or basal epithelial populations in BRCA2 mutation carriers.

(A-B) Quantification of the percentage of REV1-, POLK-, POLH-, and POLI-positive cells within the **(A)** alveolar (AV) and **(B)** basal/myoepithelial (BA) epithelial populations from the published single-cell RNA-seq dataset of $BRCA2^{mut/+}$ and $BRCA2^{+/+}$ breast epithelium. Fisher's exact test.

(C-E) Representative immunofluorescence images showing increased nuclear Pol ι (green) following BRCA2 silencing in **(C)** HMECs and **(E)** MCF7 cells. Nuclei were counterstained with DAPI (blue). Scale bars, 75 µm (HMECs) and 10 µm (MCF7).

(D-F) Quantification of nuclear Pol ι fluorescence intensity in **(D)** HMECs and **(F)** MCF7 cells following BRCA2 silencing. At least 100 cells per condition were analyzed. Data are from one biological experiment performed with three technical replicates. Red lines indicate the median; error bars denote the 95% confidence intervals. Statistical significance was determined using the Mann–Whitney U test. *$p<0.05$; **$p<0.01$; ***$p<0.001$; ****$p<0.0001$.

(G-H) Western blot analysis showing BRCA2 knockdown efficiency following siRNA-mediated BRCA2 depletion in **(G)** MCF10A and **(H)** MCF7 cells. Vinculin served as the loading control.

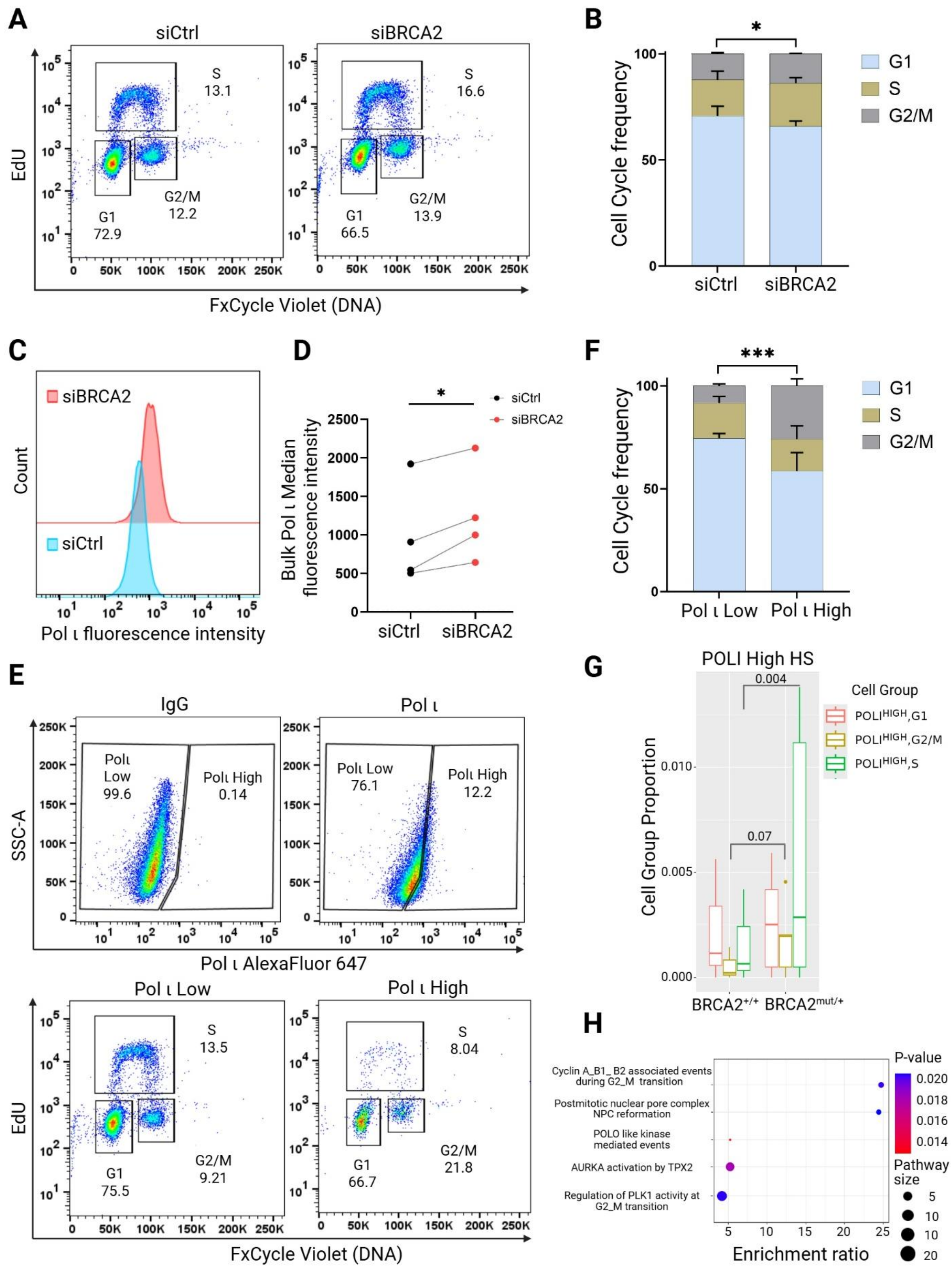
A
siCtrl
siBRCA2
S 13.1
G1 72.9
G2/M 12.2
S 16.6
G1 66.5
G2/M 13.9
EdU
FxCycle Violet (DNA)
B
Cell Cycle frequency
siCtrl
siBRCA2
G1
S
G2/M
C
siBRCA2
siCtrl
Count
Pol ι fluorescence intensity
D
Bulk Pol ι Median fluorescence intensity
siCtrl
siBRCA2
E
IgG
Pol ι
Polι Low 99.6
Polι High 0.14
Polι Low 76.1
Polι High 12.2
SSC-A
Pol ι AlexaFluor 647
Pol ι Low
Pol ι High
S 13.5
G1 75.5
G2/M 9.21
S 8.04
G1 66.7
G2/M 21.8
EdU
FxCycle Violet (DNA)
F
Cell Cycle frequency
Pol ι Low
Pol ι High
G1
S
G2/M
G
POLI High HS
Cell Group
POLI^HIGH,G1
POLI^HIGH,G2/M
POLI^HIGH,S
Cell Group Proportion
0.004
0.07
BRCA2+/+
BRCA2mut/+
H
Cyclin A_B1_ B2 associated events during G2_M transition
Postmitotic nuclear pore complex NPC reformation
POLO like kinase mediated events
AURKA activation by TPX2
Regulation of PLK1 activity at G2_M transition
P-value
Pathway size
Enrichment ratio

Supplementary Figure S2. BRCA2 deficiency increases Pol ι expression and is associated with G2/M enrichment of Pol ι-high cells.

(A) Representative flow cytometry dot plots showing cell-cycle distribution in control and BRCA2-silenced MCF10A cells.

(B) Quantification of G1, S and G2/M cell-cycle distribution following BRCA2 silencing. Data represents the mean ± SEM from three independent biological experiments. Statistical significance is shown for the G2/M comparison between siCtrl and siBRCA2 and was determined using a two-tailed unpaired Student's *t*-test. *p<0.05; **p<0.01; ***p<0.001; ****p<0.0001.

(C) Representative flow cytometry histograms showing bulk Pol ι MFI following BRCA2 silencing.

(D) Quantification of bulk Pol ι MFI from four independent biological experiments. Each dot represents one biological replicate. Statistical significance was determined using a paired Student's *t*-test. *p<0.05; **p<0.01; ***p<0.001; ****p<0.0001.

(E) Representative flow cytometry plots illustrating the gating strategy for Pol ι-low and Pol ι-high populations (top) and their corresponding cell-cycle profiles (bottom).

(F) Quantification of G1, S, and G2/M cell-cycle distribution in Pol ι-low and Pol ι-high populations. Data represents the mean ± SEM from three independent biological experiments. Statistical significance is shown for the G2/M comparison between Pol ι-low and Pol ι-high populations and was determined using a two-tailed unpaired Student's *t*-test. *p<0.05; **p<0.01; ***p<0.001; ****p<0.0001.

(G) Boxplots showing the proportions of POLI-high HS cells across cell-cycle phases in $BRCA2^{+/+}$ and $BRCA2^{mut/+}$ samples. Boxes are grouped by genotype and colored by cell-cycle phase. P values were derived using sccomp (v2.1.27), adjusting for age and patient-level effects, and were FDR-adjusted for pairwise comparisons.

(H) Dot plot showing REACTOME pathway enrichment of differentially expressed genes between POLI-high and POLI-low $BRCA2^{mut/+}$ HS cells using protein-protein interaction (PPI) network topology enrichment. The x-axis indicates the enrichment ratio (observed/expected protein-protein interaction links), the y-axis indicates the REACTOME pathway, dot size reflects gene set size, and color indicates the nominal P value.

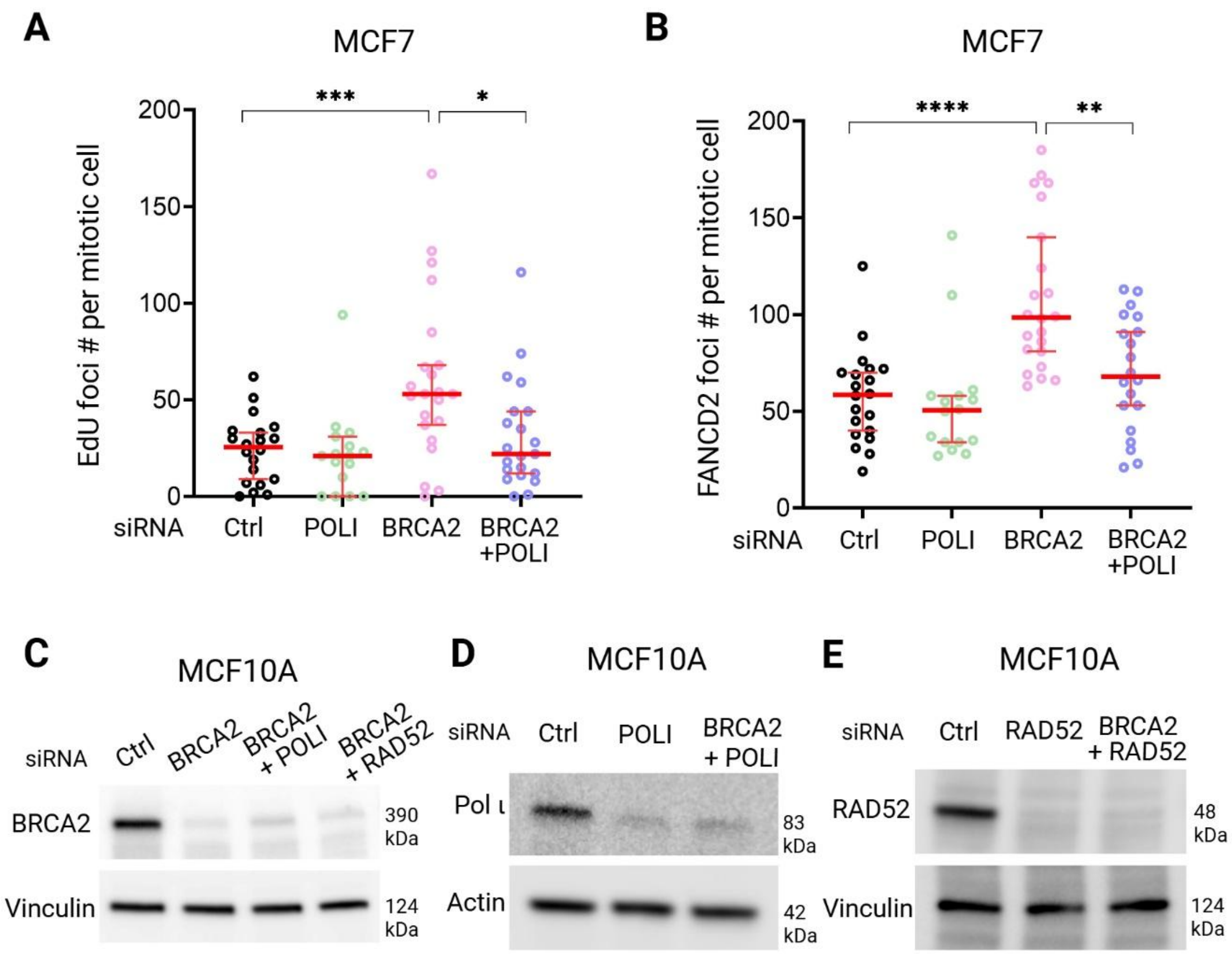

Supplementary Figure S3. Validation of BRCA2, Pol ι, and RAD52 knockdown efficiency and post-S-phase DNA synthesis readouts.

(A-B) Quantification of **(A)** EdU foci and **(B)** FANCD2 foci per mitotic cell following the indicated siRNA knockdowns in MCF7 cells. Data are from one biological experiment performed with three technical replicates. Red lines indicate the median; error bars denote the 95% confidence intervals. Statistical significance was determined using the Mann–Whitney U test. *p<0.05; **p<0.01; ***p<0.001; ****p<0.0001.

(C-E) Western blot analysis showing siRNA-mediated knockdown efficiency of **(C)** BRCA2, **(D)** POLI, and **(E)** RAD52 in MCF10A cells. Vinculin (**C** and **E**) and actin (**D**) served as loading controls.

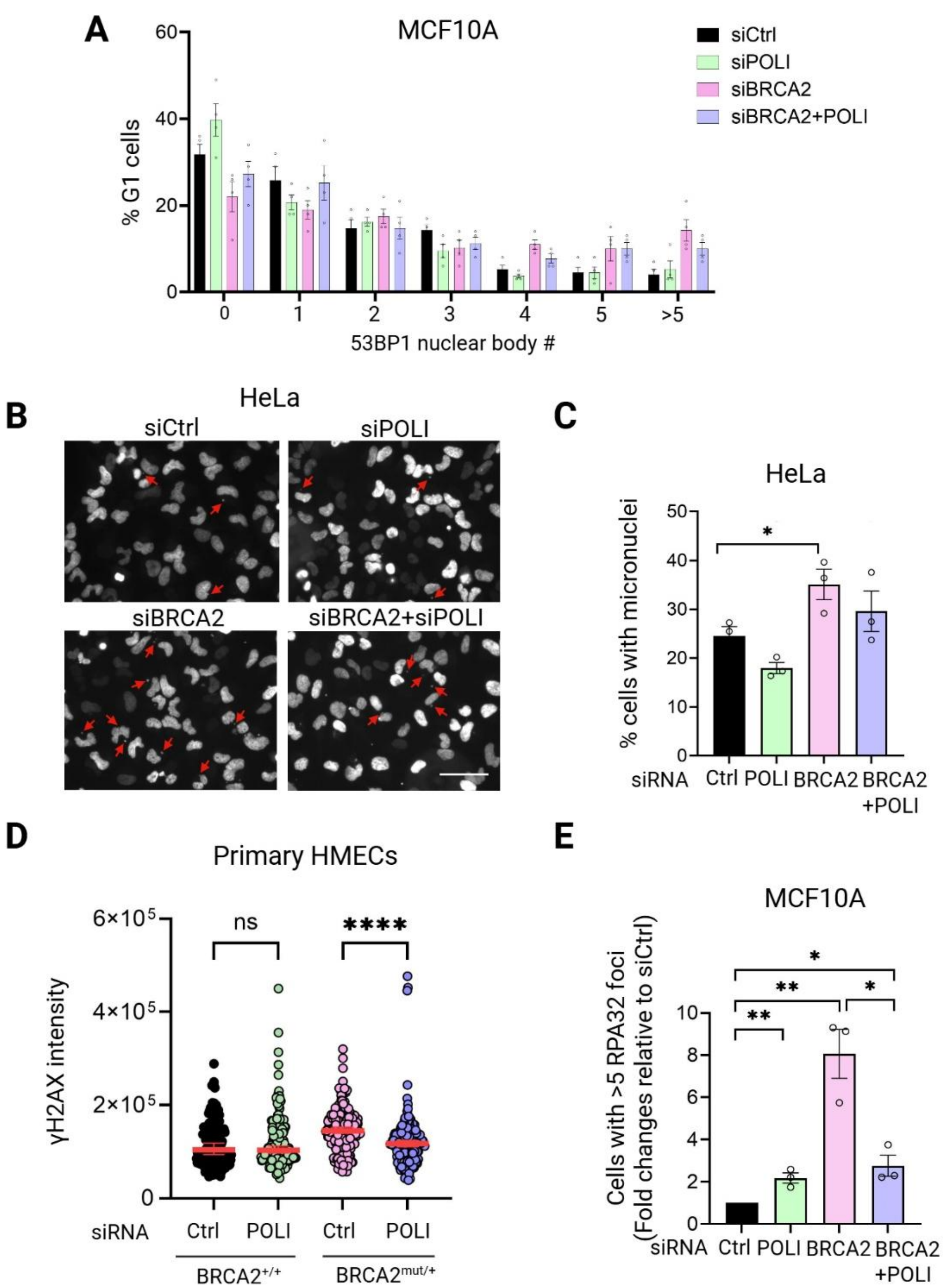

Supplementary Figure S4: Additional markers of genome instability following BRCA2 and POLI knockdown.

(A) Quantification of the number of 53BP1 NBs per Cyclin A-negative (G1) MCF10A cells following the indicated siRNA knockdowns. Data represents four independent biological experiments.

(B) Representative images of micronuclei following the indicated siRNA knockdowns in HeLa cells. Red arrows indicate micronuclei. Scale bar, 75 μm.

(C) Quantification of the percentage of micronuclei-positive cells following the indicated siRNA knockdowns in HeLa cells. Data represents three independent biological experiments.

(D) Quantification of γH2AX fluorescence intensity in primary HMECs from $BRCA2^{mut/+}$ and $BRCA2^{+/+}$ donors following POLI knockdown.

(E) Fold change in the percentage of cells containing >5 RPA32 foci relative to sictrl condition following the indicated siRNA knockdowns in MCF10A cells. Data represents three independent biological experiments.

For panels **A**, **C**, **E**, data represent the mean ± SEM. Statistical significance was determined using a two-tailed unpaired Student's *t*-test; *p<0.05; **p<0.01; ***p<0.001; ****p<0.0001. For panel **D**, each dot represents one cell; red lines indicate the median, error bars denote the 95% confidence intervals, and statistical significance was determined using the Mann–Whitney U test; *p<0.05; **p<0.01; ***p<0.001; ****p<0.0001.

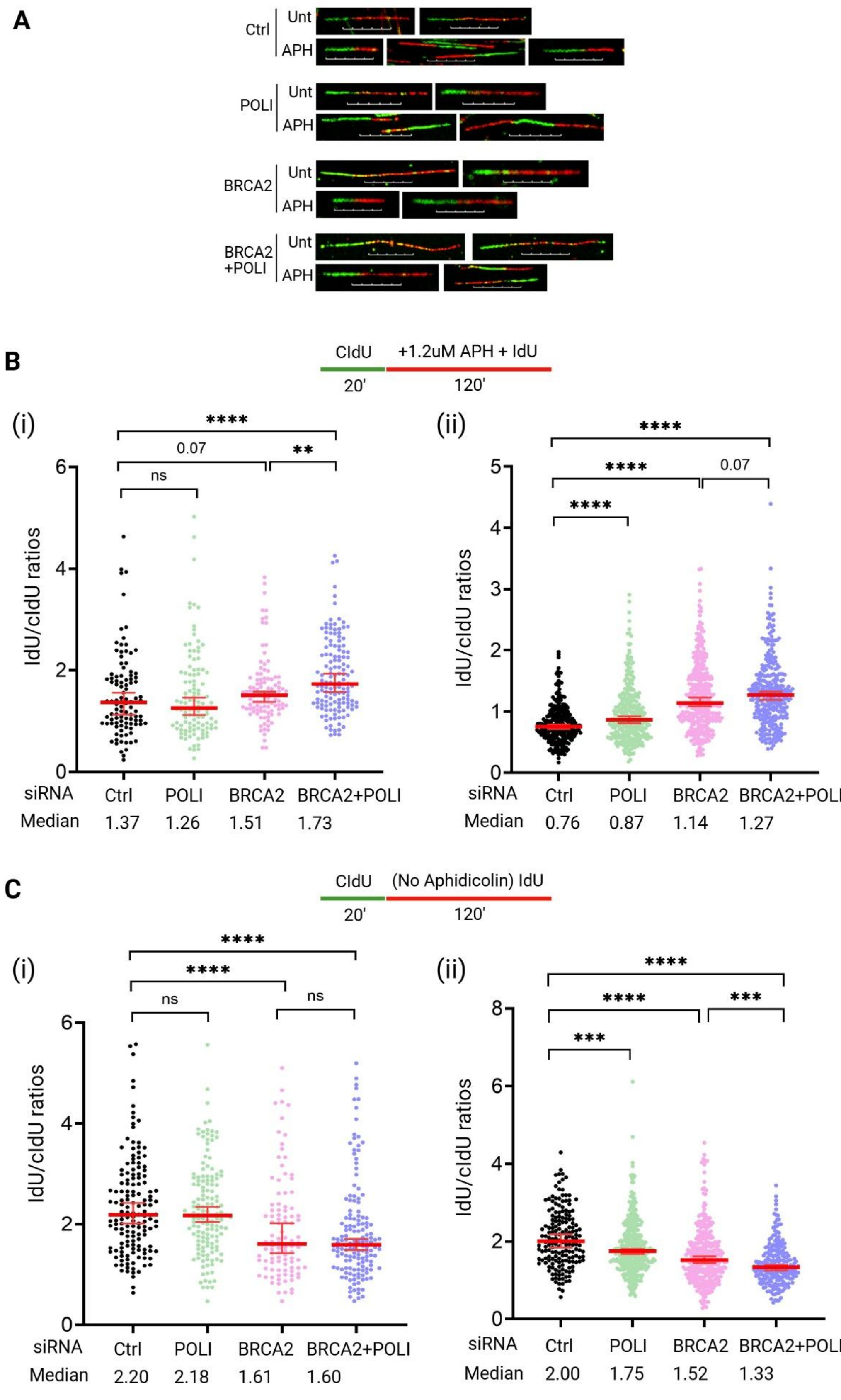
A
Ctrl
Unt
APH
POLI
Unt
APH
BRCA2
Unt
APH
BRCA2
+POLI
Unt
APH
B
CldU
+1.2uM APH + IdU
20'
120'
(i)
****
0.07
**
ns
IdU/cIdU ratios
siRNA
Ctrl
POLI
BRCA2
BRCA2+POLI
Median
1.37
1.26
1.51
1.73
(ii)
****
****
0.07
****
IdU/cIdU ratios
siRNA
Ctrl
POLI
BRCA2
BRCA2+POLI
Median
0.76
0.87
1.14
1.27
C
CldU
(No Aphidicolin) IdU
20'
120'
(i)
****
****
ns
ns
IdU/cIdU ratios
siRNA
Ctrl
POLI
BRCA2
BRCA2+POLI
Median
2.20
2.18
1.61
1.60
(ii)
****
****
***
***
IdU/cIdU ratios
siRNA
Ctrl
POLI
BRCA2
BRCA2+POLI
Median
2.00
1.75
1.52
1.33

Supplementary Figure S5. Independent validation of DNA fiber assays and baseline replication fork progression in the absence of replication stress.

(A) Representative DNA fiber tract images from the indicated siRNA knockdown conditions under untreated (Unt) or aphidicolin-treated (APH) conditions. Scale bars, 10 μm.

(B (i-ii)) Independent biological replicates of the DNA fiber assay shown in **Figure 4A**. Experimental schematics are shown above each experiment, with quantification of IdU/CIdU ratios following the indicated siRNA knockdowns shown below. Approximately 200-300 DNA fibers were analyzed per condition in each experiment.

(C (i–ii)) DNA fiber assays performed under untreated conditions (no aphidicolin). Experimental schematics are shown above each experiment, with quantification of IdU/CldU ratios following the indicated siRNA knockdowns shown below. Panel **C (i)** corresponds to the untreated condition from **Figure 4A**, whereas panel **C (ii)** corresponds to the untreated condition from **Supplementary Figure 5B (ii)**. Approximately 100–150 DNA fibers were analyzed per condition in each experiment.

For all quantifications, each dot represents one DNA fiber. Red lines indicate the median; error bars denote the 95% confidence intervals. Statistical significance was determined using the Mann–Whitney U test; *p<0.05; **p<0.01; ***p<0.001; ****p<0.0001.

Supplementary Methods

Bioinformatic analysis of public single-cell RNA-seq data

Publicly available single-cell RNA-seq data from preneoplastic breast epithelium of BRCA2 mutation carriers and wild-type controls were obtained from the GSE180878 dataset[1]. Published cell-type annotations were used to classify epithelial cells into hormone-sensing (HS), alveolar (AV), and basal/myoepithelial (BA) populations. Expression of the Y-family translesion synthesis polymerases REV1, POLH, POLK and POLI was evaluated within each epithelial population. Dot plots were generated in R using the Seurat package, with dot size representing the percentage of cells expressing each gene and color intensity indicating the average normalized expression among expressing cells. For quantification of polymerase-positive cells, cells with detectable normalized expression (>0) were classified as positive, and the proportion of positive cells was compared between BRCA2 mutation carriers and wild-type controls using Fisher's exact test.

Differential cell abundance analysis was performed using the sccomp R package (version 2.1.27)[2] while adjusting for age. Default parameters were used except bimodal_mean_variability_association = TRUE, as recommended by the package authors for single-cell data. All samples and epithelial cell types from the GSE180878 dataset[1] were included, and POLI-high/low status and cell-cycle phase were defined as described above. Cells were grouped according to genotype, patient, POLI-high/low status, and cell-cycle phase, and differential cell abundance was modeled using the formula: cell counts ~ 0 + age + genotype_POLI_cellcycle_group + (1|patient). Pairwise comparisons were performed using the default sccomp workflow, and P values were adjusted for multiple testing using the Benjamini–Hochberg false discovery rate (FDR) method.

Protein-protein interaction (PPI) network analysis

To identify biological pathways associated with POLI-high HS cells, HS cells from the GSE180878 dataset were stratified into POLI-high and POLI-low populations using a log2-normalized POLI expression cut-off at 0.875 following doublet removal and normalization with the scran[3] and scuttle[4] R packages. Differential gene expression associated with the interaction between genotype and POLI expression status was assessed using the dreamlet R package[5], with the model: expression ~ age + genotype + POLI-group + genotype x POLI-group + (1|patient).

Genes with a nominal interaction $P < 0.05$ were subjected to REACTOME pathway enrichment[6] using the ANUBIX R package[7] and protein-protein interaction (PPI) networks derived from FunCoup v6[8] (positive predictive value > 0.85) to establish gene PPI links in

the network. Enrichment was quantified as the ratio of observed to expected protein-protein interaction links (enrichment ratio). Dot plots were generated using ggplot2 R package[9], with dot size representing gene set size and color indicating the nominal P value.

To assess cell-cycle distribution, POLI-high HS cells were assigned to G1, S, or G2/M phases using Seurat R package[10]. Cell subset proportions were calculated for each patient and visualized as boxplots grouped by genotype and colored according to cell-cycle phase.

Metaphase spreads

Cells were reverse transfected with siRNAs. Forty-eight hours post-transfection, cells were treated with 0.4 µM aphidicolin (Sigma-Aldrich, Cat# 5047440001) for 24 h and arrested in metaphase by incubation with colcemid (of 0.1 µg/mL; Gibco, Cat # 15210) for 1 hour at 37 C. Cells were harvested using trysin-EDTA and subjected to 75 mM potassium chloride (Sigma, Cat # P9327) for 15 minutes at 37ºC. Cells were then fixed in freshly prepared methanol:acetic acid (3:1).

Fixed cell suspensions were dropped onto pre-cleaned glass slides (Sysmex, Cat # 024-0311-8) and air-dried under controlled humidity conditions. Slides were heat-fixed at 90ºC for 45 minutes, stained with Giemsa solution (Sigma-Aldrich, Cat # 32884), rinsed, air-dried, and mounted with coverslips (Sigma-Aldrich, Cat # 03989).

Metaphase spreads were scanned and imaged using an automated Applied Spectral Imaging system and Olympus BX63 microscope at 100x magnification. Chromosomal gaps and breaks were quantified using GenASIs software (HiBand V8.4.1, Applied Spectral Imaging) at the Cytogenetics Laboratory, Robert J.Tomsich Department of Pathology & Laboratory Medicine Institute (RT-PLMI), Cleveland Clinic.

Micronucleus assay

Cells were transfected with the indicated siRNAs and, 24 h post-transfection, treated with 0.4 µm aphidicolin for 24 hours. Following treatment, aphidicolin-containing medium was removed, cells were washed, and fresh medium containing cytochalasin-B (4.5 µg/ml, Sigma, Cat # C2743) was added for an additional 24 h to inhibit cytokinesis. Cells were then fixed with 4% paraformaldehyde and stained with DAPI. Images were acquired using EVOS M5000 and Keyence fluorescence microscopes. Micronuclei were quantified by scoring binucleated DAPI-positive cells and associated micronuclei.

siRNA sequences

Non-targeting control (siCTRL, # D-001810-10-05);

UGGUUUACAUGUCGACUAA, UGGUUUACAUGUUGUGUGA, UGGUUUACAUGUUUUCUGA, UGGUUUACAUGUUUUCCUA.

BRCA2 Human (siBRCA2, # L-003462-00-0005);

GAAACGGACUUGCUAUUUA, GGUAUCAGAUGCUUCAUUA, GAAGAAUGCAGGUUUAAUA, UAAGGAACGUCAAGAGAUA.

POLI Human (siPOLI, # L-019650-00-0005);

CCACAGUUGGUAUUAGUUA, GCACUAUGGUCGUGAGAGU, CGGGUCAUGUAUACAAUAA, GAACAUCAGGCUUUAAUAG.

References:

1. Gray, G.K., et al., A human breast atlas integrating single-cell proteomics and transcriptomics. Developmental cell, 2022. 57(11): p. 1400–1420. e7.
2. Mangiola, S., et al., sccomp: Robust differential composition and variability analysis for single-cell data. Proceedings of the National Academy of Sciences, 2023. 120(33): p. e2203828120.
3. Lun, A.T., D.J. McCarthy, and J.C. Marioni, A step-by-step workflow for low-level analysis of single-cell RNA-seq data with Bioconductor. F1000Research, 2016. 5: p. 2122.
4. McCarthy, D.J., et al., Scater: pre-processing, quality control, normalization and visualization of single-cell RNA-seq data in R. Bioinformatics, 2017. 33(8): p. 1179–1186.
5. Hoffman, G.E., et al., Efficient differential expression analysis of large-scale single cell transcriptomics data using dreamlet. BioRxiv, 2024: p. 2023.03. 17.533005.
6. Dolgalev, I., msigdbr: MSigDB Gene sets for multiple organisms in a tidy data format. R package version 7.5. 1. 2022.
7. Castresana-Aguirre, M. and E.L. Sonnhammer, Pathway-specific model estimation for improved pathway annotation by network crosstalk. Scientific Reports, 2020. 10(1): p. 13585.
8. Buzzao, D., et al., FunCoup 6: advancing functional association networks across species with directed links and improved user experience. Nucleic Acids Research, 2025. 53(D1): p. D658–D671.
9. Wickham, H., Elegant graphics for data analysis. 2016, Springer. Springer-Verlag New York. ISBN 978-3-319-24277-4. https://ggplot2.tidyverse.org.
10. Hao, Y., et al., Dictionary learning for integrative, multimodal and scalable single-cell analysis. Nature biotechnology, 2024. 42(2): p. 293–304.
